# Concentric Hybrid Nanoelectrospray Ionization‐Atmospheric Pressure Chemical Ionization Source for High‐Coverage Mass Spectrometry Analysis of Single‐Cell Metabolomics

**DOI:** 10.1002/advs.202306659

**Published:** 2024-02-15

**Authors:** Tianrun Xu, Hang Li, Peng Dou, Yuanyuan Luo, Siming Pu, Hua Mu, Zhihao Zhang, Disheng Feng, Xuesen Hu, Ting Wang, Guang Tan, Chuang Chen, Haiyang Li, Xianzhe Shi, Chunxiu Hu, Guowang Xu

**Affiliations:** ^1^ CAS Key Laboratory of Separation Science for Analytical Chemistry Dalian Institute of Chemical Physics Chinese Academy of Sciences (CAS) University of Chinese Academy of Sciences Liaoning Province Key Laboratory of Metabolomics Dalian Liaoning 116023 P. R. China; ^2^ The First Affiliated Hospital of Dalian Medical University Dalian Liaoning 116023 P. R. China; ^3^ CAS Key Laboratory of Separation Science for Analytical Chemistry Dalian Institute of Chemical Physics Chinese Academy of Sciences (CAS) University of Chinese Academy of Science Dalian Key Laboratory for Online Analytical Instrumentation Dalian Liaoning 116023 P. R. China

**Keywords:** high‐coverage, hybrid ionization source, rare cells, single‐cell metabolomics, spatially resolved metabolomics

## Abstract

High‐coverage mass spectrometry analysis of single‐cell metabolomics remains challenging due to the extremely low abundance and wide polarity of metabolites and ultra‐small volume in single cells. Herein, a novel concentric hybrid ionization source, nanoelectrospray ionization‐atmospheric pressure chemical ionization (nanoESI‐APCI), is ingeniously designed to detect polar and nonpolar metabolites simultaneously in single cells. The source is constructed by inserting a pulled glass capillary coaxially into a glass tube that acts as a dielectric barrier layer. Benefitting from the integrated advantages of nanoESI and APCI, its limit of detection is improved by one order of magnitude to 10 pg mL^−1^. After the operational parameter optimization, 254 metabolites detected in nanoESI‐APCI are tentatively identified from a single cell, and 82 more than those in nanoESI. The developed nanoESI‐APCI is successively applied to study the metabolic heterogeneity of human hepatocellular carcinoma tissue microenvironment united with laser capture microdissection (LCM), the discrimination of cancer cell types and subtypes, the metabolic perturbations to glucose starvation in MCF7 cells and the metabolic regulation of cancer stem cells. These results demonstrated that the nanoESI‐APCI not only opens a new avenue for high‐coverage and high‐sensitivity metabolomics analysis of single cell, but also facilitates spatially resolved metabolomics study coupled with LCM.

## Graphical abstract

A high‐coverage and high‐sensitivity single‐cell metabolomics method based on concentric nanoESI‐APCI hybrid ionization source is proposed, enabling simultaneous analysis of 254 polar and nonpolar metabolites. The technique is successively applied to study the metabolic heterogeneity of tumor microenvironment, the discrimination of different cells, glucose starvation‐induced metabolic perturbations in MCF7 cells, and the metabolic regulation of cancer stem cells.

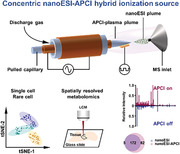

## 1 Introduction

Plenty of evidence indicated that significant inter‐cellular heterogeneity is inherent due to the stochastic regulation of gene expression and microenvironmental perturbations in biological processes.^[^ ^1^ ^]^ It is unreachable to elucidate the cellular heterogeneity through population measurements, as the average values obscure the accurate individual variations.^[^ ^2^ ^]^ Therefore, it is valuable to reveal cellular heterogeneity at the single‐cell level for faithfully reflecting the role played by individual cells in physiological processes and providing new insights into the studies of rare cell types such as circulating tumor cells (CTCs), cancer stem cells (CSCs), and primary cells. Single‐cell metabolomics has been a hotspot in recent years for it can provide instantaneous and dynamic phenotypic information.^[^ ^3^ ^]^ Compared with rapid advances in single‐cell genomics and transcriptomics, metabolome‐based analysis relatively lags due to the inherent properties of the single cell, such as picoliters magnitude of cell volume, complex structure, low content of metabolite components, and lack of amplification technology.^[^ ^4^ ^]^ Mass spectrometry (MS) has ushered in a new era of single‐cell metabolomics with its superior sensitivity, ultra‐high resolution, and rapid response.^[^ ^5^ ^]^

Nanoelectrospray ionization mass spectrometry (nanoESI‐MS), has aroused wide interest in the field of single‐cell metabolomics in recent years, due to its lower matrix effect and higher ionization effect than traditional ESI, and a series of related technologies have been derived. Induced nanoESI (InESI) owns good tolerance to matrix effects and has been utilized in metabolomics studies of mouse brain neurons and lysosomes.^[^ ^6^ ^]^ Pulsed direct current ESI‐MS (pulsed‐dc‐ESI‐MS) boosted sample economy and rich MS/MS information could be obtained from the picoliter volume of samples.^[^ ^7^ ^]^ In combination with droplet microextraction, more than 300 phospholipids have been identified in single glioblastoma cells.^[^ ^8^ ^]^ Electro‐migration and electroporation based on nanoESI can be used to analyze single cells with cell walls.^[^ ^9^ ^]^ Single‐probe and T‐probe integrating single‐cell sampling and ionization,^[^ ^10^ ^]^ enabled online and in situ single‐cell analysis, providing effective strategies for exploring cell‐cell interactions,^[^ ^11^ ^]^ cancer cell drug resistance,^[^ ^12^ ^]^ and CSCs.^[^ ^13^ ^]^ In order to satisfy the needs of practical applications, it is crucial to conduct large‐scale single‐cell metabolomics research in a high‐throughput way to obtain biologically meaningful data. Researchers have proposed several technologies, including inject printing,^[^ ^14^ ^]^ spiral capillary,^[^ ^15^ ^]^ and flow cytometry.^[^ ^16^ ^]^ The core idea of them is to separate and focus cells and introduce them individually into the MS. Our group has recently developed a technique by coupling an asymmetric serpentine channel microfluidic chip with pulsed electric field‐induced electrospray ionization‐high‐resolution MS (chip‐PEF‐ESI‐HRMS), and a throughput up to 80 cells min^−1^ was achieved, which is the highest flux reported in the literature to our knowledge.^[^ ^17^ ^]^ The system remained robust in the analysis of more than 3000 single cells continuously in a single experiment. Although some advances have been made in single‐cell metabolomics analysis, most of current studies are limited to the detection of polar metabolites with high abundance due to the ionization mechanism of nanoESI. The detection of low abundance, low ionization efficiency, and nonpolar metabolites in single cells needs innovative techniques for realizing high‐coverage single‐cell metabolomics analysis.

Various ionization modes with different mechanisms can obtain complementary information on sample composition and are conducive to expanding the detection range of metabolites. Atmospheric pressure chemical ionization (APCI) is a plasma‐based ambient soft ionization method, which is similar to the mechanism of direct analysis in real time (DART), dielectric barrier discharge ionization (DBDI) and low‐temperature plasma probe (LTP).^[^ ^18^ ^]^ In APCI, the compounds react with a large number of reactive species in the plasma plume, such as N_2_^+·^, N_4_^+·^, N_2_^*^, and H_3_O^+^ to form free radical cations or protonated ions through charge transfer, electron impact, Penning ionization, and proton transfer.^[^ ^19^ ^]^ APCI has unique advantages in ionizing weakly polar compounds and can be used as a powerful complementary tool to ESI. Therefore, the development of hybrid ionization sources with different ionization modes is helpful to improve the coverage for detecting metabolites with different polarities. Several hybrid ionization sources have been reported. For instance, the dual non‐contact nESI/nAPCI ionization source was equipped with ESI, APCI and electrophoretic separation modes for direct analysis of biological fluids and efficient detection of proteins in high‐concentration salt solutions.^[^ ^20^ ^]^ The dual ESI+APCI ionization source can be active in ESI, APCI and ESI+APCI modes by adjusting the direct current (DC) and alternating current (AC) voltage applied to the source. In combination with sampling methods including thermal desorption, laser desorption, and direct desorption/ionization, this method can be utilized for ionization and characterization of wide‐polarity metabolites in body fluid and solid samples.^[^ ^21^ ^]^ The plasma‐excited nebulizer gas‐assisted electrospray ionization (PENG‐ESI) device was established by introducing plasma into the electrospray nebulizer gas channel to detect pesticides in complex substrates with high sensitivity while maintaining good linearity, repeatability, and low matrix effects.^[^ ^22^ ^]^ The Zenobi's group introduced a combination of nanoESI and DBDI. The capillary probe was used as both cell sampling needles and nanoESI ionization source, and DBDI was used as a post‐ionization source. A total of 86 and 111 metabolites were detected from individual plant and animal cells, respectively.^[^ ^23^ ^]^ So far, most of the reported hybrid ionization sources were designed for large‐volume biological samples, and few applications based on the single‐cell level were proposed. The integration of multiple ionization modes is essential for high‐coverage single‐cell metabolomics.

Herein, we proposed a concentric nanoESI‐APCI hybrid ionization source for high‐coverage single‐cell metabolomics analysis. Compared with the single nanoESI ionization mode, the signal‐to‐noise (S/N) of the hybrid ionization mode was improved by nearly five times and the limit of detection (LOD) was also increased by 1 order of magnitude. Benefitting from multiple ionization mechanisms, the hybrid ionization source improved the coverage of metabolites in a single cell from 177 metabolites (nanoESI mode) to 254 metabolites (nanoESI‐APCI mode). The metabolic heterogeneity of tumor microenvironment (TME), the discrimination of different cancer cell types and subtypes, the metabolic perturbations to glucose starvation (GS) treatment in MCF7 cells and the metabolic differences between CSCs and non‐stem cancer cells (NSCCs) were further investigated. The practicability and feasibility of our technique for spatially resolved metabolomics, rare cell metabolomics and metabolic heterogeneity were demonstrated.

## 2 Results and Discussion

### 2.1 Configuration of the Concentric NanoESI‐APCI Hybrid Ionization Source

**Figure 1:**
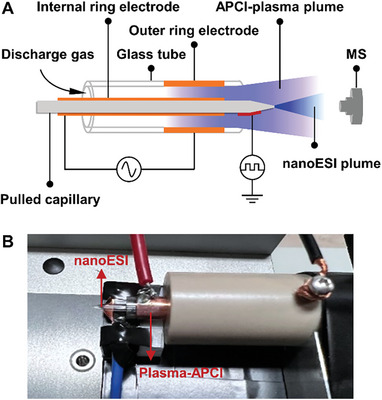
A) Schematic illustration of the concentric nanoESI‐APCI hybrid ionization source. B) Photograph of the device setup.

In the previous design, nanoESI and APCI/DBDI sources were individually connected in parallel pattern^[^ ^20^ ^]^ or tandem pattern^[^ ^23^ ^]^ as shown in Figure S1 (Supporting Information). However, the plumes of nanoESI and APCI don't overlap well in parallel pattern as well as the loss of analyte ions transport is inevitable in tandem pattern, which is particularly serious for single‐cell analysis. To overcome the shortcoming, a concentric nanoESI‐APCI hybrid ionization source was entirely redesigned in our study. The schematic illustration is shown in **Figure** **1**A. The concentric pattern does not require to transfer analyte ions between the two ionization sources and thus the nanoESI and APCI mechanisms can occur simultaneously. This hybrid ionization source is capable of two spray modes, for the pulse high voltage and AC high voltage required to generate nanoESI and APCI plasma plumes are independently controlled. (1) NanoESI mode, where the analytes in the nanoelectrospray emitter (inner diameter (I.D.) 0.86 mm, outer diameter (O.D.) 1.5 mm, with a 2–3 µm pulled tip) were charged through induced electrospray. A square‐wave pulsed high voltage with an amplitude of 5 kV and a frequency of 1 kHz was applied to a copper plate placed beneath the nanoelectrospray emitter, which could induce the pulled capillary tip to release charged droplets. (2) NanoESI‐APCI hybrid mode, in which the inner electrode (copper ring, I.D. 1.5 mm, surrounding the nanoelectrospray emitter) and the outer electrode (copper ring, I.D. 4.0 mm, surrounding the glass tube) were separated by a glass tube (I.D. 3.6 mm, O.D. 4 mm) that acted as a dielectric barrier discharge layer. When an AC voltage with an amplitude of 4 kV and a frequency of 23 kHz was applied to the inner and outer electrodes, plasma was generated inside the glass tube. Photography of the APCI‐plasma plume is shown in Figure S2 (Supporting Information). Charged droplets were generated simultaneously when a pulse high voltage was applied to the copper plate. Photography of the device setup is shown in Figure 1B.

Significant advantages of concentric nanoESI‐APCI hybrid ionization source lie in the following aspects, (1) The APCI plume effectively wraps the nanoESI plume, allowing secondary ionization of those unionized molecules in nanoESI. The secondary ionization process can further improve the sensitivity and coverage of analysis; (2) The overlap of APCI plume and nanoESI plume avoids the loss of analyte ion transport as much as possible; (3) The concentric structure design also fully considers the needs of heat control and heat dissipation. Nitrogen not only serves as a plasma discharge gas, but also effectively takes away the heat to reduce the accumulation of heat between the electrodes.

### 2.2 Effect of Discharge Gas, Gas Flow Rate, and Assistant Solvent

To examine the usefulness of the hybrid ionization source for the analysis of polar and nonpolar compounds, 10 classes of model compounds with different polarities were selected: amino acids (AAs), tricarboxylic acid cycle intermediates (TCAs), bile acids (BAs), aldehydes and ketones, nucleosides, ionic species, sterols, fatty acids (FAs), lipids and polycyclic aromatic hydrocarbons (PAHs) (Table S1, Supporting Information). The effects of discharge gas, gas flow rate, and assistant solvent on the ionization efficiency of model compounds were investigated, respectively.

#### 2.2.1 Discharge Gas

**Figure 2:**
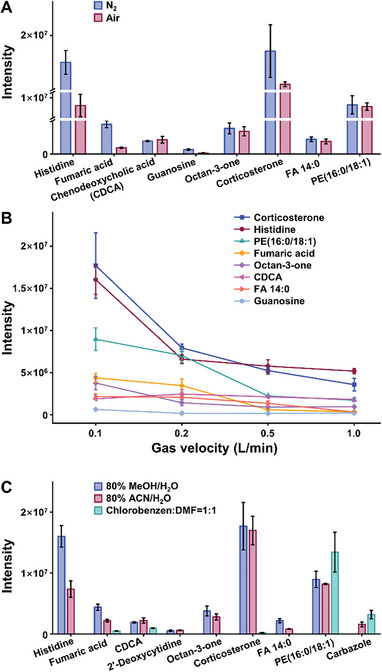
Effect of A) discharge gases, B) velocity of gas and C) assistant solvents on the intensity of representative model compounds with different polarities (*n* = 3).

The influences of two different discharge gases (N_2_, air) on the ionization efficiency are shown in **Figure** **2**A and Figure S3 (Supporting Information). For polar AAs, [M+H]^+^ and [M+Na]^+^ were observed regardless of the discharge gas used, and [M+H]^+^ was the predominant product ion. However, the intensities of [M+H]^+^ and [M+Na]^+^ were significantly increased when N_2_ was used as discharge gas. Aldehydes and ketones showed similar results as AAs. For nucleosides and ionic species, N_2_ performed slightly better than air. For compounds that are not easily ionized in the positive ion mode, such as TCAs, BAs and FAs, the main product ions in nanoESI‐APCI mode were [M]^+•^, and the absolute highest intensities were seen when using N_2_ as the discharge gas. For nonpolar sterols and lipids, N_2_ also exhibited higher ionization efficiency due to increased intensities of [M+H]^+^ and [M+Na]^+^, and [M+H]^+^ was the predominant product ion. Therefore, N_2_ was selected as the discharge gas for its better performance.

#### 2.2.2 Gas Flow Rate

The effects of different discharge gas flow rates (0.1 L min^−1^, 0.2 L min^−1^, 0.5 L min^−1^, 1.0 L min^−1^) on the ionization efficiency of the model compounds were also assessed (Figure 2B; Figure S4, Supporting Information). It can be seen that the signal intensity of both polar and non‐polar compounds decreased with the increase of N_2_ flow rate. To ensure high ionization efficiency, a flow rate of 0.1 L min^−1^ was chosen.

#### 2.2.3 Assistant Solvent

Previous research^[^ ^24^ ^]^ has shown that a suitable solvent can boost the ionization of PAHs. Herein, the effects of three different solvents (80% methanol (MeOH)/H_2_O, 80% acetonitrile (ACN)/H_2_O, chlorobenzene/N,N‐dimethylformamide (DMF) = 1:1) on the ionization efficiency of polar and nonpolar compounds were evaluated (Figure 2C; Figure S5, Supporting Information). For AAs, aldehydes and ketones, nucleosides and ionic species with strong polarity, the absolute signal intensities were much higher when dissolved in 80% MeOH/H_2_O and 80% ACN/H_2_O than in chlorobenzene/DMF = 1:1. For TCAs, BAs and FAs, highest ionization efficiency was seen when dissolved in 80% MeOH/H_2_O. For the nonpolar sterols, the signal intensities were highest when the assistant solvent was 80% MeOH/H_2_O, followed by 80% ACN/H_2_O, while no obvious product ions were detected in hybrid mode when the assistant solvent was chlorobenzene/DMF = 1:1. For nonpolar lipids, the ionization efficiencies of three solvents were similar, with chlorobenzene/DMF = 1:1 slightly higher than 80% MeOH/H_2_O and 80% ACN/H_2_O. PAHs, which are highly nonpolar and rarely found in cells, were also used to assess the ability of concentric nanoESI‐APCI hybrid ionization source to analyze nonpolar compounds. Using chlorobenzene/DMF = 1:1 as an assistant solvent led to higher ionization efficiency. Ultimately, 80% MeOH/H_2_O was selected as an assistant solvent considering the ionization efficiency of compounds with different polarity comprehensively.

### 2.3 NanoESI‐APCI Versus NanoESI: Ionization Efficiency and Sensitivity

**Figure 3:**
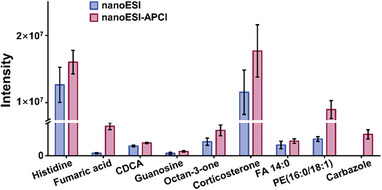
MS signal intensity of representative model compounds with different polarities in nanoESI and nanoESI‐APCI modes (*n* = 3).

Based on the above optimized conditions, the comparisons of the ionization efficiency of 10 classes of model compounds with different polarities in nanoESI mode and nanoESI‐APCI hybrid mode were displayed in **Figure** **3** and Figure S6 (Supporting Information). In nanoESI mode, the product ions of polar AAs were [M+H]^+^ and [M+Na]^+^, among which [M+H]^+^ produced by the proton transfer reaction was the main product ion. In hybrid mode, the intensity of [M+H]^+^ slightly decreased and the intensity of [M+Na]^+^ slightly increased. For nucleosides and aldehydes and ketones, the main product ions were [M+H‐H_2_O]^+^ and [M+H]^+^, respectively. These outcomes indicated that turning on APCI helps to improve ionization efficiency. For TCAs, BAs and FAs, the analyte ions were presented as radical cations [M]^+•^. These carboxylic metabolites were usually poor ionization in the positive ion mode of ESI source. Higher absolute signal intensities were seen in hybrid mode. For nonpolar sterols and lipids, the product ions were [M+H]^+^ and [M+Na]^+^ with [M+H]^+^ dominating. The responses of all product ions increased when APCI was turned on. For strong nonpolar PAHs that are not easily ionized in nanoESI mode, the plasma generated by the hybrid ionization source can ionize PAHs to [M]^+•^ in the positive ion mode through a series of ionization reactions, such as charge transfer, electron impact and Penning ionization. The possible reaction mechanism involved in the ionization process can be seen in the Supporting Information.

S/N, LOD and linearity were used as metrics for evaluating the sensitivity of the ionization source. Ten model compounds with a concentration of 1 ng mL^−1^ were used to compare the S/N acquired in both modes. The results showed that the signal intensity in nanoESI‐APCI mode was 1.81–7.31 times of that in nanoESI mode, and 1.85–4.84 fold increase in S/N was obtained in hybrid mode (**Table** **1**). Concerning the LOD, nanoESI‐APCI mode achieved lower LOD than nanoESI for all model compounds (Table S2, Supporting Information). Taking histidine as an example, the LOD of hybrid ionization was only 0.01 ng mL^−1^ while that of nanoESI was 0.1 ng mL^−1^. Overall, the LOD of nanoESI‐APCI mode is 2–10 times lower than that of nanoESI mode. Calibration curves were established using a series of model compound solutions with different concentrations to evaluate the linear range. The hybrid mode exhibited a wider linear range than nanoESI mode and demonstrated good linearity with R^2^ > 0.99 (Table S2, Supporting Information). The better performance of the hybrid mode indicated that the nanoESI‐APCI ionization source was a sensitive analytical tool that was qualified for mining low‐abundance metabolite information in single cells to obtain a richer single‐cell metabolic profile.

**Table 1:** Signal intensity and S/N acquired in both modes for 1 ng mL^−1^ model compounds.

Metabolite	Formula	m/z	Signal intensity			S/N		
			nanoESI	nanoESI‐APCI	nanoESI‐APCI/ nanoESI	nanoESI	nanoESI‐APCI	nanoESI‐APCI/ nanoESI
Histidine	[M+H]^+^	156.0768	3.49×10^4^	2.55×10^5^	7.31	13.45	65.12	4.84
Fumaric acid	[M]^+·^	116.0705	1.61×10^5^	6.91×10^5^	4.29	64.04	185.25	2.89
CDCA	[M]^+·^	392.2875	2.77×10^5^	1.18×10^6^	4.26	9.24	37.78	4.09
Guanosine	[M+H‐H_2_O]^+^	266.2472	1.33×10^4^	5.61×10^4^	4.22	4.72	14.79	3.14
4‐Hydroxy‐3‐methoxy‐cinnamaldehyde	[M+H]^+^	179.0703	9.45×10^3^	4.07×10^4^	4.31	3.52	10.47	2.97
Choline chloride	[M]^+^	146.1177	1.08×10^6^	2.33×10^6^	2.16	27.54	61.60	2.24
Corticosterone	[M+Na]^+^	369.2036	8.64×10^3^	2.46×10^4^	2.85	3.04	5.93	1.95
FA 14:0	[M]^+·^	228.1958	2.39×10^5^	4.32×10^5^	1.81	7.57	14.00	1.85
PC 18:0/18:1	[M+H]^+^	788.6151	7.63×10^4^	2.08×10^5^	2.73	2.55	6.22	2.44
Carbazole	[M]+·	167.0791	7.60×10^4^	1.93×10^5^	2.54	3.02	8.93	2.96

### 2.4 Performance of the NanoESI‐APCI Hybrid Ionization Source in Single‐Cell Metabolite Analysis

**Figure 4:**
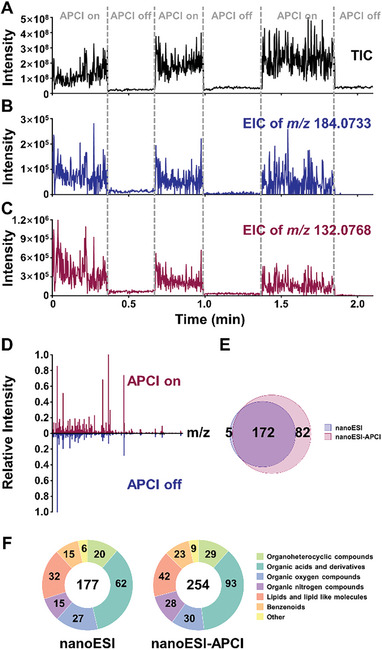
Metabolite analysis in a representative single MCF7 cell analyzed by nanoESI‐APCI hybrid ionization source. A) TIC, B) EIC of phosphorylcholine (m/z 184.0733), and C) EIC of creatine (m/z 132.0768) acquired in nanoESI and nanoESI‐APCI modes. D) Mass spectrum acquired in APCI on/off modes. E) Venn diagram of detected metabolite numbers in both modes. F) Classifications of detected metabolites in both modes.

The performance of the hybrid ionization source was further evaluated through single‐cell metabolomics analysis. Taking MCF7 cells as an example, after sampling partial cytoplasm (Figure S7 and Video S1, Supporting Information), metabolomics analysis of a single MCF7 cell was performed using the hybrid ionization source. As presented in the total ion chromatogram (TIC) (**Figure** **4**A), higher signal intensity could be achieved with the APCI switched on. The peaks at m/z 184.0733 and 132.0768 in the extracted ion chromatogram (EIC) were assigned to phosphocholine and creatine, respectively, which are regarded as markers of ionization of single‐cell contents.

Figure 4B,C displayed that the variations of the signal intensity of EIC for phosphocholine and creatine were in good agreement with the TIC. It can be seen from Figure 4D that richer spectral information could be obtained when the APCI was switched on. Benefited from the high sensitivity, secondary mass spectra of metabolites can also be obtained, which is useful for the accurate identification of metabolites (Figure S8, Supporting Information). In total, in the positive ion mode, based on the accurate mass, secondary mass spectra and the LC‐MS analysis results of bulk cells, 177 metabolites were detected in nanoESI mode (Table S3, Supporting Information), while 254 metabolites in nanoESI‐APCI mode (Table S3, Supporting Information), of which 172 metabolites were detected in both modes and 82 metabolites were detected only in hybrid mode (Figure 4E). Figure 4F summarized the classifications of detected metabolites in both modes, including many important functional metabolites, such as amino acids, acylcarnitines, organic acids, and lipid‐like molecules. In addition, nonpolar organic heterocyclic and benzenoid metabolites were also detected. About 70% of the annotated metabolites were validated by traditional LC‐MS analysis of population cells (Table S3, Supporting Information). The nanoESI‐APCI hybrid source also has unique advantages in capturing key functional metabolites. The intensity comparison of the 8 key functional metabolites in the TCA pathway in two modes (Figure S9, Supporting Information) showed that the hybrid mode can significantly improve the detection sensitivity and ensure that key metabolites can be effectively detected and analyzed, thus providing more in‐depth and comprehensive metabolomic insights. In addition, the intensity of non‐polar metabolites represented by FAs in hybrid mode is significantly improved compared with nanoESI mode, demonstrating the potential of the nanoESI‐APCI hybrid source in improving the detection ability of non‐polar metabolites (Figure S10, Supporting Information). The performance of our method and other related four works reported in the previous studies was summarized in Table S4 (Supporting Information) and Figure S11 (Supporting Information) for comparison. These results suggested that the nanoESI‐APCI hybrid ionization source is powerful for analyzing single‐cell metabolome.

### 2.5 Practical Applications of NanoESI‐APCI Hybrid Ionization Source in Cell Discrimination

To show the practicability, the method developed was respectively applied to study the spatial differentiation of tumor metabolism, the heterogeneity of different cancer cell types and subtypes, the metabolic perturbations to GS treatment in MCF7 cells and the metabolic dysregulation of prostate cancer stem cells.

#### 2.5.1 Tumor Heterogeneity Research Based on Spatially Resolved Metabolomics

**Figure 5:**
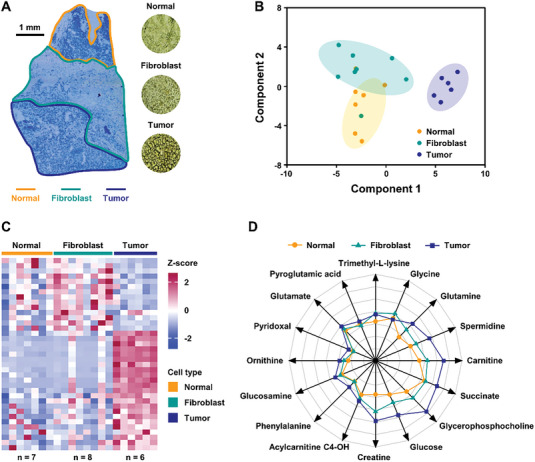
Performance of nanoESI‐APCI hybrid ionization source for the discrimination of tissue microregions. A) The Nissl staining image of the HCC tissue section. B) PCA score plot based on differentiated features (*p* < 0.05). C) Heatmap of significantly changed features with *p* < 0.05 for the discrimination of 7 normal tissue microregions, 8 fibroblast tissue microregions and 6 tumor tissue microregions. D) Logarithmic radar charts of mean relative intensities of 16 characteristic metabolites.

It is well recognized that tumor heterogeneity is attributed to complex and diverse genetic or epigenetic alterations and evolution.^[^ ^25^ ^]^ Spatially resolved metabolomics helps to understand the metabolic reprogramming of the TME by characterizing the spatial distribution of metabolites. Spatial distribution information is frequently lost in traditional LC‐MS‐based studies in which metabolites are extracted from tissue homogenates. Moreover, the averaged results obscure the spatially resolved information of metabolites in heterogeneous tumor tissues. Ambient ionization techniques, represented by desorption electrospray ionization (DESI),^[^ ^26^ ^]^ airflow‐assisted desorption electrospray ionization (AFADESI),^[^ ^27^ ^]^ nanospray desorption electrospray ionization (nanoDESI),^[^ ^28^ ^]^ and laser ablation electrospray ionization (LAESI),^[^ ^29^ ^]^ have been developed to obtain molecular images directly on the sample surface. A series of groundbreaking insights have been reaped in clinical fields of tumor boundary recognition,^[^ ^27^, ^30^ ^]^ antitumor drug assessment,^[^ ^31^ ^]^ therapeutic target discovery^[^ ^32^ ^]^ and prognosis evaluation.^[^ ^33^ ^]^ In addition, the combination of laser capture microdissection (LCM) technology and mass spectrometry detection is also one of the strategies for spatially resolved metabolomics. Since its inception, LCM has proven itself to be capable of microdissecting microregions of interest based on a highly focused and precisely controlled laser beam and collecting them for offline metabolomics analysis.^[^ ^34^ ^]^ Here, a human hepatocellular carcinoma (HCC) tissue sample was obtained by surgical resection and frozen sections were analyzed by LCM combined with nanoESI‐APCI hybrid ionization source to discover the heterogeneous spatial distribution of metabolites in TME. The laser “cut and drop” sampling process is shown in Figure S12 (Supporting Information). Nissl staining of tissue sections in **Figure** **5**A revealed considerable heterogeneity in tumor morphology. According to histologic types, HCC tissue can be divided into paracancerous normal tissues, fibroblast tissues and tumor tissues, all of which have obvious tumor‐peripheral boundaries. The principal component analysis (PCA) score plot based on microregion‐specific mass spectra showed no obvious clustering between normal tissues and fibroblast tissues, but a clear separation was observed between tumor tissues and normal tissues (including paracancerous normal tissues and fibroblast tissues), suggesting that phenotypically different tissue microregions have distinguishable metabolic profiles and distribution (Figure 5B). The heatmap of characteristic features (*p* < 0.05) displayed that metabolites exhibited heterogeneous distribution associated with tumor histomorphology (Figure 5C). The content distribution of 16 differential metabolites in three microregions is shown in Figure 5D. Most metabolites showed higher enrichment in tumor microregions than that in normal microregions, which might be related to the higher metabolic activity of tumor tissues. This is also a reflection of the high degree of intra‐tumor heterogeneity, which might be attributed to spatial variation in metabolic activity during tumor development.

The expression of glucose in tumor microregions was significantly higher than that in normal microregions because the large uptake of glucose supplies HCC's metabolic activities.^[^ ^35^ ^]^ Increased pyroglutamic acid, glutamine, and glutamate were detected in tumor microregions, which is consistent with previous studies showing that tumor cells have a high glutamine uptake and utilization to support tumor survival and proliferation under antioxidant stress.^[^ ^36^ ^]^ Carnitine in the tumor tissue undergone significant and specific enrichment compared to normal microregions, and trimethyl‐lysine was slightly upregulated, which may demonstrate increased degradation of lysine in tumor tissues. The significant reprogramming of carnitine, a key metabolite in energy metabolism, including fatty acid β‐oxidation, indicated the high energy requirements of tumor cell proliferation. There is substantial evidence that down‐regulated succinate dehydrogenase (SDH) gene activities result in significantly increased succinate levels in HCC,^[^ ^35^, ^37^ ^]^ which was further validated in our spatially resolved metabolomics results. The accumulation trend of phenylalanine from normal microregions to tumor microregions supported the previous conclusions that the increase of phenylalanine is indeed associated with the development of HCC, and it was defined as a prognostic marker to assess overall survival.^[^ ^38^ ^]^

#### 2.5.2 Classification of Different Cancer Cell Types and Subtypes

To test the usefulness of the method for cell discrimination, three different types of cancer cells were distinguished by unsupervised t‐distributed stochastic neighbor embedding (t‐SNE) analysis based on the single‐cell metabolic profiles, including 43 MCF7 cells, 39 97H cells and 50 PC3 cells. As shown in Figure S13A (Supporting Information), three types of cancer cells formed distinct clusters with significant differences. Enriched metabolites with *p* < 0.05 among three groups were represented in the heatmap (Figure S13B, Supporting Information). The intensities of metabolites were similar in the same cancer cell types but varied significantly among different cancer cell types. Figure S13C (Supporting Information) visualizes the KEGG pathway analysis based on differential metabolites. Heterogeneity of three cancer cell types was strongly associated with alanine, aspartate and glutamate metabolism, TCA cycle, glycine, serine and threonine metabolism, arginine and proline metabolism, pyruvate metabolism and arginine biosynthesis, as they all exhibited lower *p*‐values and greater pathway impacts.

Two different prostatic cancer (PCa) cell subtypes (50 PC3 cells and 34 DU145 cells) were analyzed to further verify the practicality of the method. The t‐SNE algorithm can effectively distinguish different cancer cell subtypes based on single‐cell metabolic profiles (Figure S14A, Supporting Information). The volcano plot in Figure S14B (Supporting Information) compared metabolic profiles of two PCa cell subtypes. Statistically significant features were highlighted in red (upregulated) and blue (downregulated). The heatmap displayed statistically significant metabolites for the discrimination of subtypes of PCa cells (Figure S14C, Supporting Information). It also showed that the abundances of metabolites in the same cancer subtype were comparable but remarkably varied in different cancer cell subtypes. KEGG pathway enrichment analysis showed that alanine, aspartate and glutamate metabolism, TCA cycle, glycine, serine, and threonine metabolism had greater influences on the metabolic heterogeneity of PCa cell subtypes (Figure S14D, Supporting Information).

#### 2.5.3 GS‐Induced Metabolic Perturbations in MCF7 Cells

Nutrient restriction is a key factor affecting cellular physiological status and metabolic pathways, especially for tumor cells with active metabolic processes. Exploring their behavior under nutrient restriction is of great help to gain insight into the adaptive mechanism and metabolic reprogramming of tumor cells under nutritional stress. As a key nutrient source for cell growth and energy metabolism, glucose deficiency can greatly change the metabolic characteristics of cells. To study the effect of GS on metabolites in MCF7 cells, glucose‐free medium was utilized to culture MCF7 cells for 24 h followed by single‐cell metabolomics analysis. In the comparison studies, the same measurements were conducted using normally cultured MCF7 cells. The t‐SNE result demonstrated the significant clustering and separation between GS treatment and control groups, indicating significant metabolic changes triggered by GS (Figure S15A, Supporting Information). The volcano plot in Figure S15B (Supporting Information) reflected markedly different metabolic profiles in the glucose‐deficient environment, which showed obvious metabolic differences compared to the control group. The heatmap of differential metabolites (*p* < 0.05) showed obvious metabolic disorders after GS treatment (Figure S15C, Supporting Information). To further explore perturbations of metabolic pathways induced by GS, differential metabolites were mapped to biochemical pathways for enrichment analysis, as shown in Figure S15D (Supporting Information). Alanine, aspartate and glutamate metabolism, TCA cycle, glycine, serine and threonine metabolism, pyruvate metabolism and arginine biosynthesis were the most perturbed pathways, in which the metabolites mainly showed a decreasing trend (Figure S15E, Supporting Information). In the absence of glucose, glycolysis was restricted, resulting in a reduction in pyruvate, which directly affected the downregulation of TCA cycle intermediates. Changes in amino acid metabolism may indicate adaptive strategies to tackle the lack of energy and carbon sources. These changes reflected complex metabolic adaptations of cells in facing nutritional stress and provided a new perspective for understanding the metabolic reprogramming of tumor cells under nutrient restriction.

#### 2.5.4 Metabolite Heterogeneity Between CSCs and NSCCs

**Figure 6:**
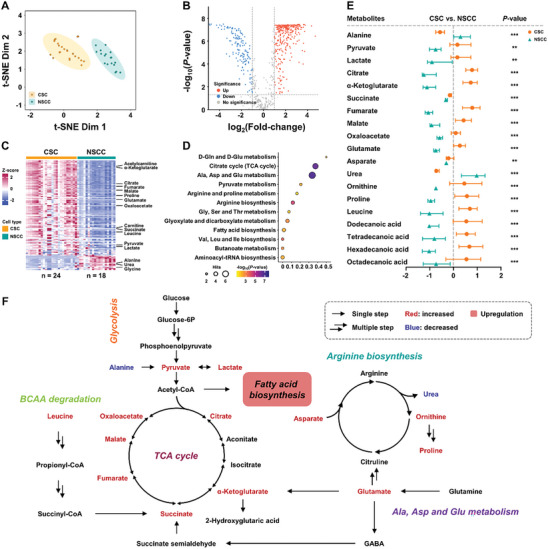
Performance of nanoESI‐APCI hybrid ionization source for the discrimination of CSCs and NSCCs. A) t‐SNE cluster analysis with 95% confidence ellipses illustrating the overall difference of metabolomics profiles between 24 CSCs and 18 NSCCs. B) Volcano plot showing correlations between *p*‐value and fold‐change for all metabolites of CSCs compared to those of NSCCs. Characteristic metabolites are highlighted in red (upregulated) and blue (downregulated). C) Heatmap of significantly changed metabolites with *p* < 0.05 for the discrimination of CSCs and NSCCs. Color indicates *z*‐scores of metabolites. D) KEGG metabolic pathways differentially regulated in CSCs compared to NSCCs. Each circle was colored by the ‐log_10_(*p*‐value) and the size was correlated to the number of matched metabolites. E) *Z*‐score plot of 19 representative metabolites with differential abundance in CSC/NSCC cells. Data are presented as median with interquartile range and points are colored by assigned cell type. *: 0.001 < *p* < 0.05, **: 0.001 < *p* < 0.0001, ***: *p* < 0.0001. F) Simplified schematic depicting TCA cycle activation in CSCs.

CSCs are rare types of cells that play a vital role in the development, recurrence, and metastasis of cancer. Traditional studies for CSCs metabolomics are limited by the small number of cells. The single‐cell technique based on the nanoESI‐APCI hybrid ionization source is a promising tool for metabolomics studies on rare cells such as CSCs. We utilized PCa (CWR‐22Rv1) CSCs as the model system, and conventional CWR‐22Rv1 as controls for NSCCs to investigate the overall metabolic differences at the single‐cell level. To clearly visualize the differences in metabolic profiles between CSCs and NSCCs, t‐SNE was applied to visualize group discrimination and reduce the dimensionality of the complex metabolic profiles to a two‐dimensional plane. The outcome of t‐SNE cluster analysis with 95% confidence ellipses based on the metabolomic profiles obtained from 24 CSCs and 18 NSCCs was displayed in **Figure** **6**A. It can be seen that CSCs were well separated from NSCCs, indicating significant metabolic differences between the two groups. The volcano plot in Figure 6B displayed the correlations between *p*‐value and fold‐change (FC) of metabolic profiles. Significant changes in the level of 483 metabolic features (|log_2_FC| > 1, *p* < 0.05) were observed, reflecting obvious metabolic differences of CSCs versus NSCCs. The heatmap showed the relative abundances of differential metabolites (*p* < 0.05) for discriminating CSCs and NSCCs (Figure 6C), indicating significant heterogeneity in single‐cell metabolic profiles between CSCs and NSCCs. Special attention was paid to metabolites with statistically significant differences (*p* < 0.05) between CSCs and NSCCs in t‐SNE analysis, 24 of them were only detectable in nanoESI‐APCI. Figure S16 (Supporting Information) shows that these metabolites exhibit significantly different distribution patterns in CSCs and NSCCs, confirming the unique value of the nanoESI‐APCI hybrid source in revealing metabolic heterogeneity. Then we used the differential metabolites for enrichment analysis of KEGG pathways to identify the metabolic pathways that own strong influence on cell heterogeneities and observed significant enrichment of citrate cycle (TCA cycle), alanine, aspartate and glutamate metabolism, and arginine biosynthesis (Figure 6D).

We further investigated the regulation of energy metabolism centering on TCA cycle. Altogether, a total of 19 representative differential metabolites associated with these metabolic pathways were screened out. Their relative abundances (*z*‐score) in CSCs and NSCCs are shown in Figure 6E. We also mapped these metabolites to TCA cycle and related pathways (Figure 6F) to reveal the regulation network underlying PCa progression at the single‐cell level. TCA cycle intermediate metabolites, including citrate, α‐ketoglutarate (α‐KG), succinate, fumarate, malate, and oxaloacetate were markedly accumulated in CSCs. The significant upregulation of glutamate, branched chain amino acid (BCAA) represented by leucine and the enrichment of metabolites related to arginine biosynthesis and fatty acid biosynthesis pathway were also found. These results revealed that CSCs and NSCCs have distinct energy metabolism pathways at the single‐cell level. Many previous studies have reported activated TCA cycle in PCa,^[^ ^39^ ^]^ which is consistent with our results at the single‐cell level. Based on PCa tissues and matched adjacent normal tissues, our group previously integrated metabolomics and transcriptomics analysis, and systematically elucidated the potential underlying metabolic reprogramming and regulatory mechanisms from the perspective of gene‐ metabolite regulatory networks.^[^ ^39^, ^40^ ^]^ Significant enrichment of TCA cycle intermediate metabolites was observed in tumor tissues, which benefited from the following several metabolic pathways.^[^ ^39b^ ^]^ First, the enhanced pyruvate supplemented TCA cycle. Second, leucine provided substrates for TCA cycle through BCAA degradation pathway. Additionally, glutamine anaplerosis promoted TCA cycle by providing α‐KG. To our knowledge, it is the first time that accelerated TCA cycle in PCa has been verified at the single‐cell level. In this research, the application of the nanoESI‐APCI hybrid source was critical to ensure the effective detection and analysis of key metabolites in the TCA cycle. The technology significantly improves the detection sensitivity, demonstrating its unique advantages in capturing key functional metabolites. It not only deepens our understanding of the distinct energy metabolism pathways between CSCs and NSCCs, but also provides more accurate and comprehensive metabolomic data, further strengthening our comprehension of the regulatory network of PCa progression at the single‐cell level.

## 3 Conclusions

In conclusion, a novel concentric nanoESI‐APCI hybrid ionization source was established to provide a convenient and efficient solution for high‐coverage and high‐sensitivity analysis of single‐cell metabolomics and spatially resolved metabolomics. The creative hybrid ionization source has significant advantages in providing higher ionization efficiency and analytical sensitivity for both polar and nonpolar metabolites. The higher S/N and lower LOD indicated that the nanoESI‐APCI hybrid ionization source is qualified for mining metabolite information of low abundance, poor ionization efficiency, and weak polarity in single cells to obtain a richer single‐cell metabolic profile. The hybrid source showed excellent performance in single‐cell metabolomics analysis and 254 metabolites were preliminarily identified from a single cell. The differentiation of three types of cancer cell lines and PCa cell subtypes demonstrated the universality of the proposed technique for cellular heterogeneity analysis. The monitoring of GS‐induced metabolic perturbations in MCF7 cells reflected the potential application of the technique in mechanism exploration. In combination with LCM, differential regulation of metabolites associated with HCC tumor histomorphology was validated, proving that our method can conduct spatially resolved metabolomics analysis for precise tumor typing. The method was further applied to the study of metabolic characteristics of CSCs demonstratively, and activated TCA cycle in PCa at the single‐cell level was first reported. It means that our technique can be further extended to the metabolomics studies of other rare cells and provide a new paradigm for comprehensive and in‐depth insights into pathogenesis and the discovery of new functional biomarkers as therapeutic targets. The nanoESI‐APCI system possessed the advantages of high coverage, high sensitivity, and rich information for single cells detection, which is expected to promote the development of single‐cell metabolomics and spatially resolved metabolomics.

## 4 Experimental Section

### Reagents and Materials

Detailed information was provided in the Supporting Information.

### Sample Pretreatment

Forty standard compounds from 10 different classes including AAs, TCAs, BAs, aldehydes, and ketones, nucleosides, ionic species, sterols, FAs, lipids, and PAHs were prepared as model compounds. Detailed information on the 40 model compounds is provided in Table S1 (Supporting Information). Detailed descriptions on sample pretreatment were provided in the Supporting Information.

### Concentric NanoESI/APCI Hybrid Ionization Source

The schematic diagram of a homemade concentric nanoESI‐APCI hybrid ionization source is shown in Figure S1 (Supporting Information), which is different from parallel pattern^[^ ^20^ ^]^ and tandem pattern^[^ ^23^ ^]^ reported in previous studies. The hybrid ionization source is composed of available commercial components and eliminates the need for fine machining. In brief, a pulled glass capillary (I.D. 0.86 mm, O.D. 1.5 mm, with a 2–3 µm pulled tip) was inserted into a coaxial glass tube (I.D. 3.6 mm, O.D. 4 mm), keeping the tip of the fused glass capillary extending 5 mm from the glass tube. The glass tube was utilized as the dielectric barrier discharge layer. A high voltage and high‐frequency AC voltage of 4 kV_PP_ at a frequency of 23 kHz was applied between the inner ring copper electrode (I.D. 1.5 mm, surrounding the pulled glass capillary) and the outer ring copper electrode (I.D. 4.0 mm, surrounding the glass tube), which will drive the gas flowing in the glass tube to be ignited to generate plasma. Nitrogen was used as discharge gas and fed into the glass tube from the back end at a flow rate of 0.1 L min^−1^. The gas flow rate was monitored in real‐time using a flow meter (LZM‐4T). The AC power supply was provided by a customized power module (ZNOG 0503A N202). Photography of the APCI‐plasma plume is shown in Figure S2 (Supporting Information). The pulsed power supply was applied to a copper plate beneath the pulled tip of the glass capillary to induce inductive electrospray. The pulse high voltage was provided by a homemade adjustable square‐wave pulsed high voltage power supply, the details can be found in a previous publication.^[^ ^17^ ^]^ Briefly, a square‐wave pulse voltage was generated by a waveform generator and fed to a fast high‐voltage transistor switch. By controlling the DC high voltage module, a square‐wave pulsed high voltage with an amplitude of 5 kV, a frequency of 1 kHz, and a duty cycle of 50% can be output. The entire device is mounted on a home‐built three‐dimensional adjustment platform with the pulled tip of the glass capillary aligned with the MS inlet at a distance of 10 mm.

### Cell Culture and Sampling

According to the optimum culture protocol recommended by ATCC, all cell lines were cultured in a 10 cm diameter dish containing 10% fetal bovine serum (FBS), 1% penicillin‐streptomycin solution and dulbecco's modified eagle medium (DMEM), and placed in an incubator at 37 °C and 5% CO_2_. Detailed descriptions on cell culture and sampling were provided in the Supporting Information.

### CSC and NSCC Culture

The CSCs were obtained by microsphere culture method. Detailed information was provided in the Supporting Information.

### HCC Tissue Sample Collection and Processing

One HCC tissue sample was collected by surgical resection, having been approved by the local Ethical Review Board of the First Affiliated Hospital of Dalian Medical University (Dalian, China, PJ‐KS‐KY‐2023‐77). The patient provided written informed consent. Detailed descriptions on HCC sample collection and processing were provided in the Supporting Information.

### Sampling and Pretreatment of Tissue Microregions from Tissue Sections

The commercial LCM system (Leica LMD7000, Leica Microsystems, Wetzlar, Germany) was used for microdissection of tissue sections, based on a highly focused and precisely controlled laser beam dissecting microregions of interest and collecting them for later analysis.^[^ ^34^ ^]^ Detailed descriptions on sampling and pretreatment of tissue microregions from tissue sections were provided in the Supporting Information.

### Mass Spectrometry

The Q Exactive‐HF MS (Thermo Fisher Scientific, San Jose, CA, USA) was utilized for full scan MS data acquisition in the positive ion mode. The calibration solution was used to calibrate the mass axis before measurements to ensure high mass accuracy. The mass spectra were acquired across the mass range of 70–1050 with a resolution for MS1 of 60 000 at m/z 200. The MS was operated with a capillary temperature of 275 °C. The S‐Lens RF level was set to 50 and one microscan was applied. The automatic gain control (AGC) target of 1e5 and maximum injection time (IT) of 100 ms were applied for full MS scan.

### LC‐MS Analysis of Population Cells

When 80%−90% of the 10 cm culture dishes were covered by cells, cells were washed three times by phosphate buffer saline (PBS) and 1 mL cold MeOH/H_2_O (8:2, v/v) containing internal standards was added. The cells were scraped off the culture dishes and transferred to 2 mL Eppendorf centrifuge tubes for 2 min sonication. After equilibration for 10 min, the samples were centrifuged at 14 000 rpm for 15 min at 4 °C, and 600 µL supernatant was lyophilized and resuspended in 50 µL ACN/H_2_O (1:3, v/v) for subsequent LC‐MS analysis. Detailed information was provided in the Supporting Information.

### Data Processing and Analysis

A homemade Python script was developed to process single‐cell metabolomics raw data, peak alignment, stable feature ion screening, metabolites identification and machine learning. Detailed information was provided in the Supporting Information.

## Conflict of Interest

The authors declare no conflict of interest.

## Author Contributions

T.X. and H.L. contributed equally to this work. Dr. P.D. contributed to sampling of tissue microregions from tissue sections by LCM. Y.L. contributed to CSC and NSCC culture. S.P. prepared frozen sections of HCC tissue. D.F., X.H., and T.W. contributed to data acquisition and statistical analyses. Z. Zhang contributed to the design of the device. H.M. and Prof. G.T. provided HCC tissue sample. C.C. and Prof. H.L. critically revised the manuscript for important intellectual content and approved the version to be published. Prof. G.X., Prof. C.H., and Prof. X.S. are the guarantors of this work and, as such, had full access to all the data in the study and take responsibility for the integrity of the data and the accuracy of the data analysis.

## Supporting information

Supporting Information

Supplemental Video 1

## Data Availability

The data that support the findings of this study are available in the supplementary material of this article.
