# Supplementary material for: Concentric Hybrid Nanoelectrospray Ionization‐Atmospheric Pressure Chemical Ionization Source for High‐Coverage Mass Spectrometry Analysis of Single‐Cell Metabolomics
Source: Adv Sci (Weinh). 2024 Feb 15;11(16):2306659. doi: 10.1002/advs.202306659 (PMC11040340; doi:10.1002/advs.202306659)
Supplement: Supplementary file 1 — Supporting Information [file ADVS-11-2306659-s002.pdf]

ADVANCED SCIENCE
Open Access

Supporting Information

for *Adv. Sci.*, DOI 10.1002/advs.202306659

Concentric Hybrid Nanoelectrospray Ionization-Atmospheric Pressure Chemical Ionization Source for High-Coverage Mass Spectrometry Analysis of Single-Cell Metabolomics

Tianrun Xu, Hang Li, Peng Dou, Yuanyuan Luo, Siming Pu, Hua Mu, Zhihao Zhang, Disheng Feng, Xuesen Hu, Ting Wang, Guang Tan, Chuang Chen, Haiyang Li, Xianzhe Shi, Chunxiu Hu* and Guowang Xu**

Supporting Information

Concentric Hybrid Nanoelectrospray Ionization-Atmospheric Pressure Chemical Ionization Source for High-Coverage Mass Spectrometry Analysis of Single-Cell Metabolomics

Tianrun Xu[1,2,3‡], Hang Li[1,3‡], Peng Dou[1,3], Yuanyuan Luo[1,2,3], Siming Pu[1,2,3], Hua Mu[5], Zhihao Zhang[1,2,4], Disheng Feng[1,2,3], Xuesen Hu[1,2,3], Ting Wang[1,3], Guang Tan[5], Chuang Chen[1,2,4], Haiyang Li[1,2,4], Xianzhe Shi[1,2,3*], Chunxiu Hu[1,2,3*], Guowang Xu[1,2,3*]

1 CAS Key Laboratory of Separation Science for Analytical Chemistry, Dalian Institute of Chemical Physics, Chinese Academy of Sciences, Dalian, 116023, People's Republic of China

2 University of Chinese Academy of Sciences, Beijing 100049, People's Republic of China

3 Liaoning Province Key Laboratory of Metabolomics, Dalian, 11023, People's Republic of China

4 Dalian Key Laboratory for Online Analytical Instrumentation, Dalian, 11023, People's Republic of China

5 The First Affiliated Hospital of Dalian Medical University, Dalian, 11023, People's Republic of China

‡ These authors contributed equally to this work.

[*] Correspondence authors:

Prof. Dr. Guowang Xu, CAS Key Laboratory of Separation Sciences for Analytical Chemistry, Dalian Institute of Chemical Physics, Chinese Academy of Sciences, Dalian 116023, China. Tel. / Fax: 0086-411-84379530. E-mail: xugw@dicp.ac.cn.

Prof. Dr. Chunxiu Hu, CAS Key Laboratory of Separation Science for Analytical Chemistry, Dalian Institute of Chemical Physics, Chinese Academy of Sciences, Dalian 116023, China. E-mail: hucx@dicp.ac.cn.

Prof. Dr. Xianzhe Shi, CAS Key Laboratory of Separation Science for Analytical Chemistry, Dalian Institute of Chemical Physics, Chinese Academy of Sciences, Dalian 116023, China. E-mail: shixianzhe@dicp.ac.cn.

Table of Contents

Experimental section ········ S4
The possible reaction mechanism involved in the ionization process ········ S8

Supporting Figures:

Figure S1. Schematic of previously reported nanoESI-APCI/DBDI hybrid ionization sources and this work. ········ S10
Figure S2. Photograph of the APCI-plasma plume. ········ S11
Figure S3. Effect of plasma gases (N_2, air) on the intensity of 9 classes of model compounds with different polarities. ········ S11
Figure S4. Effect of velocity of N_2 (0.1 L/min, 0.2 L/min, 0.5 L/min 1.0 L/min) on the intensity of 9 classes of model compounds with different polarities. ········ S12
Figure S5. Effect of assistant solvents (80% MeOH/H_2O, 80% ACN/H_2O, Chlorobenzene: DMF=1:1) on the intensity of 10 classes of model compounds with different polarities. ········ S13
Figure S6. Analysis of 10 classes of model compounds with different polarities in nanoESI mode and nanoESI-APCI mode. ········ S14
Figure S7. Photograph of an actual sampling of a single MCF7 cell. ········ S15
Figure S8. MS2 spectra obtained from a single MCF7 cell. ········ S15
Figure S9. Intensity comparisons of 8 key metabolites in nanoESI mode and nanoESI-APCI mode. · S16
Figure S10. Intensity comparisons of 8 non-polar metabolites in nanoESI mode and nanoESI-APCI mode. ········ S16
Figure S11. Metabolite analysis in a single PANC-1 cell analyzed by the hybrid ESI-DBDI source proposed by Zenobi's group. ········ S17
Figure S12. The laser 'cut and drop' sampling process. ········ S17
Figure S13. Discrimination results of cancer cell types. ········ S18
Figure S14. Discrimination results of PCa cell subtypes. ········ S19
Figure S15. The influence of GS treatment on metabolites of MCF7 cells. ········ S20
Figure S16. The outcome of t-SNE cluster analysis of metabolites with statistically significant differences ($p < 0.05$) between 24 CSCs and 18 NSCCs. ········ S21

Supporting Tables:

Table S1. Detailed information of 10 classes of model compounds. ········ S22
Table S2. LOD acquired in both modes for different model compounds. ········ S24

Table S3. Assigned metabolites from a single cell in nanoESI and nanoESI-APCI modes. ············ S25
Table S4. Performance comparison between related studies and ours. ····································· S44

References ··· S45

Experimental Section

Reagents and materials

High-performance liquid chromatography (HPLC)-grade acetonitrile (ACN) and methanol (MeOH) were purchased from Merck (Darmstadt, Germany). Ultrapure water (H_2O) was produced by the Milli-Q system (Millipore, Billerica, MA). Chlorobenzene was purchased from Damao Chemical Reagent Factory (Tianjin, China). Ammonium formate (AmFa) and formic acid were purchased from ACMEC Biochemical (Shanghai, China). Valine was purchased from Fisher Scientific (San Jose, CA, USA). Tyrosine, serine, histidine, lactic acid, fumaric acid, chenodeoxycholic acid (CDCA), lithocholic acid, uridine, inosine, guanosine, cytidine, acetylcholine chloride and 4-hydroxy-3-methoxy-cinnamaldehyde were obtained from Sigma-Aldrich (St. Louis, MO, USA). Malic acid was purchased from Chem Service (Herrnsheimer Hauptstr, Germany). Succinic acid and fatty acid (FA) 20:1 were purchased from Fluka (Steinheim, Germany). Cholic acid (CA) was purchased from Toronto Research Chemicals (Toronto, Canada). 2'-Deoxycytidine was purchased from Tokyo Chemical Industry (Tokyo, Japan). 2,4-Dimethylbenzaldehyde, (E)-3-phenylprop-2-enal, octan-3-one and N,N-dimethylformamide (DMF) were purchased from Macklin (Shanghai, China). Choline chloride and corticosterone were purchased from J&K Scientific (Beijing, China). 11β-hydroxytestosteron, 5α-androstane-3β,17β-diol and testosterone were purchased from International Laboratory (San Francisco, USA). Progesterone and androstenedione were purchased from Dr.Ehrenstorfer GmbH (Augsburg, Germany). FA 14:0, FA 16:0 and FA 18:0 were purchased from Nu-Chek Prep (Elysian, MN, USA). Phosphatidylethanolamine (PE) 16:0/18:1, phosphatidylcholine (PC) 18:0/18:1, PC (18:0/18:2), sphingomyelin (SM) d18:1/18:0 and SM (d18:1/24:0) were purchased from Avanti Polar Lipids (Alabaster, AL, USA). Carbazole, fluorine and methylfluorene were purchased from AflaAesar (Aksaray, Turkey).

Breast cancer cells (MCF7), prostate cancer cells (DU145, PC3, CWR-22Rv1) and liver cancer cells (97H) were purchased from the Council cell bank of Typical Culture Preservation, CAS (Shanghai, China). Dulbecco's modified eagle medium (DMEM), DMEM/F12 (1:1), phosphate buffer saline (PBS), Accutase and penicillin-streptomycin were purchased from Gibco Life Technologies (Carlsbad, CA, USA). Fetal bovine serum (FBS) was prepared from Biological Industries (Kibbutz Beit Haemek, Israel). B27 was purchased from Thermo Fisher Scientific (San Jose, CA, USA). Epidermal growth factor (EGF) and basic fibroblast growth factor (bFGF) were purchased from Petrotech.

Borosilicate glass capillaries (BF150-86-10, inner diameter (I.D.) 0.86 mm, outer diameter (O.D.) 1.5 mm) were purchased from Sutter Instrument (Novato, CA, USA). Polyethylene naphthalate (PEN) membrane glass slides were purchased from Leica Microsystems (Wetzel, Germany).

Sample pretreatment

Forty standard compounds from 10 different classes including amino acids (AAs), tricarboxylic acid cycle (TCAs), bile acids (BAs), aldehydes and ketones, nucleosides, ionic species, sterols, FAs, lipids and polycyclic aromatic hydrocarbons (PAHs) were prepared as model compounds. Detailed information on the 40 model compounds is provided in Table S1. The 10 classes of model compounds were prepared in 80% MeOH/H_2O, 80% ACN/H_2O and chlorobenzene/DMF = 1:1 at the concentration of 10 μg/mL, respectively. To evaluate S/N and LOD of two ionization modes, 10 representative model compounds were selected and dissolved in 80% MeOH/H_2O, in which histidine at 1 μg/mL, fumaric acid at 1 μg/mL, chenodeoxycholic acid at 5 μg/mL, guanosine at 1 μg/mL, 4-hydroxy-3-methoxy-cinnamaldehyde at 1 μg/mL, choline chloride at 0.1 μg/mL, corticosterone at 1 μg/mL, FA 14:0 at 5 μg/mL, PC 18:0/18:1 at 5 μg/mL and carbazole at 5 μg/mL. The solution was then gradually diluted.

Cell culture and sampling

According to the optimum culture protocol recommended by ATCC, all cell lines were cultured in a 10 cm diameter dish containing 10% FBS, 1% penicillin-streptomycin solution and DMEM, and placed in an incubator at 37 °C and 5% CO_2. For the glucose starvation experiment of MCF7 cells, after removing the DMEM medium, the cells were washed 3 times by PBS, and the cell medium was changed to glucose-free DMEM supplemented with 10% FBS and cultured for 24 h. Cells were washed 3 times by PBS to remove the medium that could interfere with mass spectra. Finally, the dish was filled with PBS and placed under an inverted microscope stage (CKX53, Olympus, Tokyo, Japan) to select targeted cells and monitor the sampling process.

Borosilicate glass capillary tube (I.D. 0.86 mm, O.D. 1.5 mm) was pulled into the micropipette with a tip of ~ 2 μm opening using P-1000 puller (Sutter Instruments, Novato, CA, USA). The parameters are as follows: heat = 546, pull = 0, velocity = 19, delay = 1, and pressure = 600. The pulled glass capillary was fixed on a metal holder, which attached to a 3-dimensional translation manipulator platform (MP-225, Sutter Instrument, Novato, CA, USA). The capillary was carefully inserted into the cell, and negative pressure was applied through an air pump connected to the back end of the metal holder. The cytoplasm was sucked for 90 s. After sampling, the capillary was inserted into the device coaxially to construct the concentric nanoESI-APCI hybrid ionization source. The sampling image of the single cell is shown in Figure S7. A visualized cell sampling process is provided in Video S1.

CSC and NSCC culture

The CSCs were obtained by microsphere culture method. CWR-22Rv1 cells were seeded in a 6-well ultralow attachment plate with serum-free sphere medium, and CSCs would proliferate and form suspended cell spheres. Sphere medium consisted of DMEM/F12 (1:1, v/v), 20 ng/mL bFGF, 20 μL/mL

B27 and 20 ng/mL EGF. After 7-10 days, CSCs were filtered and collected with a 100 μm cell sieve. Then, cells were digested with 1 mL Accutase at 37 °C water bath for 10 min, and 2 mL sphere medium was added to terminate the digestion. Next, cells were transferred to a 5 mL centrifuge tube for a 2 min centrifugation at 100 *g*. Finally, CSCs were washed twice with PBS and resuspended in PBS for subsequent single-cell sampling.

For comparison, we utilized the regular CWR-22Rv1 cells cultured in DMEM/F12 (1:1, v/v) containing 10% FBS as the model of NSCCs. The culture process was detailed in the "Cell culture and sampling" section.

Human hepatocellular carcinoma (HCC) tissue sample collection and processing

One HCC tissue sample was collected by surgical resection, having been approved by the local Ethical Review Board of the First Affiliated Hospital of Dalian Medical University (Dalian, China, PJ-KS-KY-2023-77). The patient provided written informed consent. The diameter of the tissue sample was 1-2 cm. The HCC tissue was flash-frozen in liquid nitrogen for 20 s after resection, then transferred to cryogenic vials and stored at -80 °C until sectioned to a 10-μm-thick frozen section at -20 °C using a Leica CM1950 cryostat microtome (Leica Biosystems Nussloch GmbH, Wetzlar, Germany). The 10-μm-thick frozen sections were mounted onto 2.0 μm PEN membrane glass slides. Nine adjacent frozen sections were prepared, one of which was stained by cresyl violet acetate. Before the microdissection, the frozen slices were dried in a vacuum for ~30 min. The Nissl staining image of HCC tissue is illustrated in Figure 5A, it can be observed that HCC tissue section had three histologic types: paracancerous normal tissues, fibroblast tissues and tumor tissues.

Sampling and pretreatment of tissue microregions from tissue sections

The commercial laser capture microdissection (LCM) system (Leica LMD7000, Leica Microsystems, Wetzlar, Germany) was used for microdissection of tissue sections, based on a highly focused and precisely controlled laser beam dissecting microregions of interest and collecting them for later analysis[1]. After laser beam cutting of the targeted microregions outlines drawn using the LMD7000 mapping tool, microregions were recovered by gravity-assisted dropping of the section into a collection container (the capture cap of a 0.2 mL centrifuge tube), located below the sample substrate. Figure S12 demonstrates the 'cut and drop' sampling process. Five μL extraction solvent (80% MeOH/H_2O, 10 mM AmFa, 10% formic acid) was added to the collection tube and ultrasonication was performed for 30 min using a non-contact ultrasonic crusher (MX-96A, XiaoMeiChaoSheng, Kunshan, China) to extract metabolites from the microregions. The concentric nanoESI/APCI hybrid ionization source was then used for subsequent analysis.

LC-MS analysis of population cells

When 80%-90% of the 10 cm culture dishes were covered by cells, cells were washed 3 times by PBS and 1 mL cold MeOH/H_2O (8:2, v/v) containing 12 internal standards was added. The chemical names and concentration information were as follows: carnitine C2:0-d3 (0.1 μg/mL), carnitine C10:0-d3 (0.29 μg/mL), lyso-phosphatidylcholine 19:0 (0.75 μg/mL), tryptophan-d5 (4.25 μg/mL), phenylalanine-d5 (3.6 μg/mL), choline-d4 (2 μg/mL), CA-d4 (0.5 μg/mL), CDCA-d4 (0.5 μg/mL), leucine-enkephalin (2.5 μg/mL), glutamic acid-d3 (4 μg/mL), FFA C16:0-d3 (2.5 μg/mL), FFA C18:0-d3 (2.5 μg/mL). The cells were scraped off the culture dishes and transferred to 2 mL Eppendorf centrifuge tubes for 2 min sonication. After equilibration for 10 min, the samples were centrifuged at 14,000 rpm for 15 min at 4 °C, and 600 μL supernatant was lyophilized and resuspended in 50 μL ACN/H_2O (1:3, v/v) for subsequent LC-MS analysis.

The ACQUITY ultra-high performance liquid chromatography system (UHPLC, Waters, Milford, MA, USA) coupled with the Q Exactive-HF MS was used for LC-MS analysis of population cells. The columns for the positive and negative ion modes were ACQUITY UPLC BEH C8 (100 mm × 2.1 mm, 1.7 μm, Waters, Milford, MA, USA) and ACQUITY UPLC HSS T3 (100 mm × 2.1 mm, 1.8 μm, Waters, Milford, MA, USA), respectively. The column temperature was 50 °C, the flow rate was 0.35 mL/min, and the injection volume was set to 5 μL. In the positive ion mode, the mobile phases A and B were H_2O with 0.1% formic acid and ACN with 0.1% formic acid, respectively. The elution gradient started with 5% B and maintained for 1 min. Then it was increased linearly to 100% B within 23 min and was kept for 4 min. The gradient was changed back to the initial gradient within 0.1 min and was held for 1.9 min to equilibrate the column. The total run time was 30 min. In the negative ion mode, the mobile phases A and B were H_2O and MeOH, respectively, both containing 6.5 mM NH_4HCO_3. The initial gradient was 2% B and was kept for 1 min, then linearly increased to 100% B within 17 min and maintained for 4 min. The gradient was back to 2% B at 22.1 min and held for 2.9 min. The total run time was 25 min.

The MS was operated with a spray voltage of 3.5 kV in the positive ion mode and 3.0 kV in the negative ion mode. The capillary temperature was 300 °C, the flow rate of the sheath gas and auxiliary gas were set to 45 and 10 (in arbitrary units), respectively. The S-lens RF level was set as 50. The mass range was 85-1250 m/z. The resolutions of 120,000 and 30,000 at m/z 200 were separately set for full MS scan and data dependent MS/MS (ddMS2). The AGC target of 3e6 and maximum IT of 200 ms were applied for full MS scan. While the values were 1e5 and 50 ms in ddMS2 settings. The top 10 ions with the highest abundance per full MS scan were selected for MS/MS acquisition. The normalized collision energies (NCE) were 15, 30, 45 eV, respectively, and the dynamic exclusion time was 7.0 s.

Metabolites were identified based on retention time, accurate mass and MS/MS spectra using HMDB, METLIN, mzCloud, MoNA and home-built databases.

Data processing and analysis

A homemade Python script was developed to process single-cell metabolomics raw data, peak alignment, stable feature ion screening, metabolites identification and machine learning. Raw MS data were recorded with Xcalibur software (v2.2, Thermo Fisher Scientific, San Jose, CA, USA), and exported as CSV files including m/z, intensity and S/N. Phosphorylcholine (m/z 184.0733) and creatine (m/z 132.0768) were used as markers for the detection of single-cell events to extract all ion signals associated with the ionization of single-cell contents. Signals with S/N greater than 3 and the detection rate in all cell events greater than 50% were selected as stable characteristic ions for subsequent analysis. After the ion intensities were normalized to total ion intensity, peak alignment was performed between different single cell samples. The metabolites were tentatively assigned according to the accurate mass comparison of the database (mass error < 5 ppm) and MS/MS spectra obtained by liquid chromatography-mass spectrometry (LC-MS) analysis of population cells. Databases include Human Metabolome Database (HMDB, http://www.hmdb.ca), METLIN Metabolite Database (https://metlin.scripps.edu/), mzCloud (https://www.Mzcloud.org/), MassBank of North America (MoNA, https://mona.fiehnlab.ucdavis.edu/) and home-made database OSI-SMMS. A machine learning algorithm based on t-distributed stochastic neighbor embedding (t-SNE) was utilized to reduce the dimensionality of complex metabolic datasets to a two-dimensional plane. Principal component analysis (PCA) was performed using SIMCA-P software (v13.0, Ume trics, Umea, Sweden). The Mann-Whitney *U* test (M-W test) and Kruskal-Wallis *H* test (K-W test) were used to evaluate the statistical significance level (*p*-value) of single-cell metabolic profiles between two or three different cell types. SPSS software (v25.0, IBM, Armonk, USA) was used to conduct the evaluation, and $p < 0.05$ was considered to be statistically significant. Volcano plots were drawn using Origin software (Origin2023, Northampton, Massachusetts, USA). Heatmap visualization was performed using Multi Experiment Viewer software (MeV, v4.9.0, Dana-Farber Cancer Institute, MA, USA) to show the expression level of single-cell metabolites among different types of cell lines. Kyoto Encyclopedia of Genes and Genomes (KEGG) pathway analysis was conducted on MetaboAnalyst website (https://www.metaboanalyst.ca/).

The possible reaction mechanism involved in the ionization process

The ionization process involves two key reactions, namely the charge transfer reaction and the proton transfer reaction.

The charge transfer reaction mainly uses $N_2^{+\bullet}$ and $O_2^{+\bullet}$ to generate $[M]^{+\bullet}$.[2] The reaction formulas involved are as follows:

$$N_2 + e^- \rightarrow N_2^{+\cdot} + 2e^-$$

$$N_2^{+\cdot} + 2N_2 \rightarrow N_4^{+\cdot} + N_2$$

$$N_4^{+\cdot} + O_2 \rightarrow O_2^{+\cdot} + 2N_2$$

$$M + O_2^{+\cdot} \rightarrow M^{+\cdot} + O_2$$

The proton transfer reaction mainly uses H_3O^+ to generate $[M+H]^+$.[2c, 3] The reaction formulas involved are as follows:

$$N_2 + e^- \rightarrow N_2^{+\cdot} + 2e^-$$

$$N_2^{+\cdot} + 2N_2 \rightarrow N_4^{+\cdot} + N_2$$

$$N_4^{+\cdot} + H_2O \rightarrow 2N_2 + H_2O^{+\cdot}$$

$$H_2O^{+\cdot} + H_2O \rightarrow H_3O^+ + OH^\cdot$$

$$H_3O^+ + M \rightarrow [M+H]^+ + H_2O$$

Supporting Figures

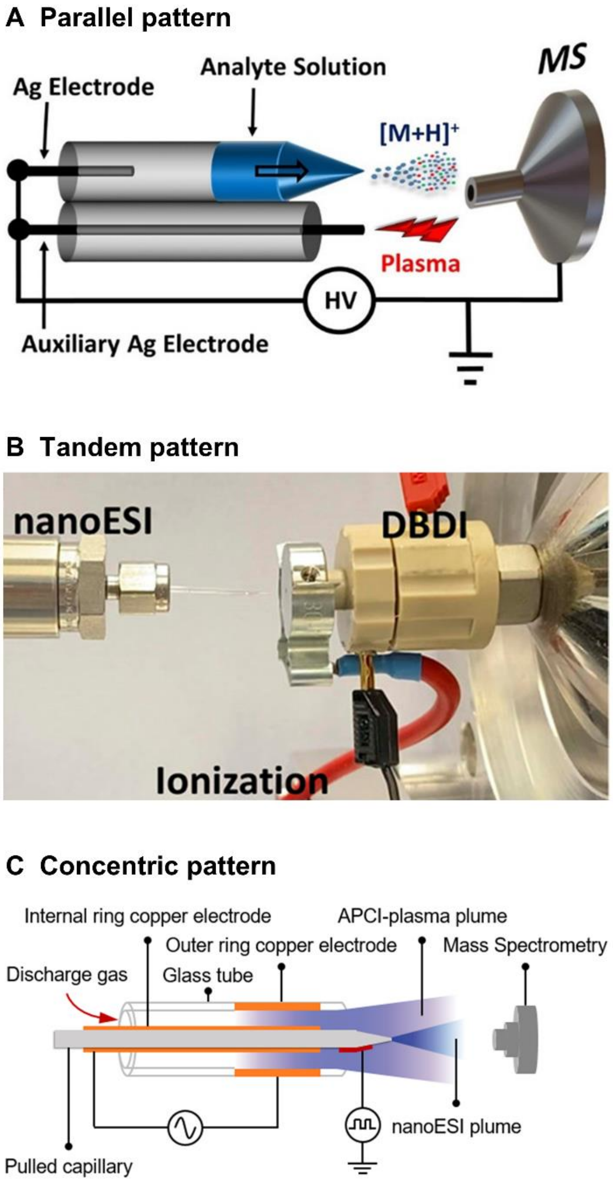

Figure S1. Schematic of previously reported nanoESI-APCI/DBDI hybrid ionization sources and this work. (A) Schematic of parallel nESI/nAPCI. Reproduced with permission.[4] Copyright (2019) American Chemical Society. (B) Schematic of tandem nanoESI-DBDI. Reproduced with permission.[5] Copyright (2022) American Chemical Society. (C) Schematic of concentric nanoESI-APCI hybrid ionization source.

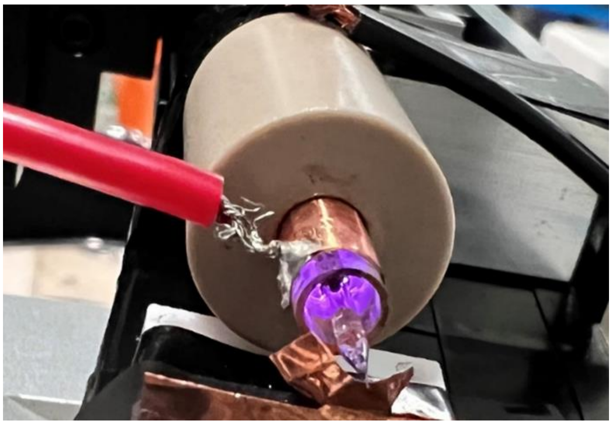

Figure S2. Photograph of the APCI-plasma plume.

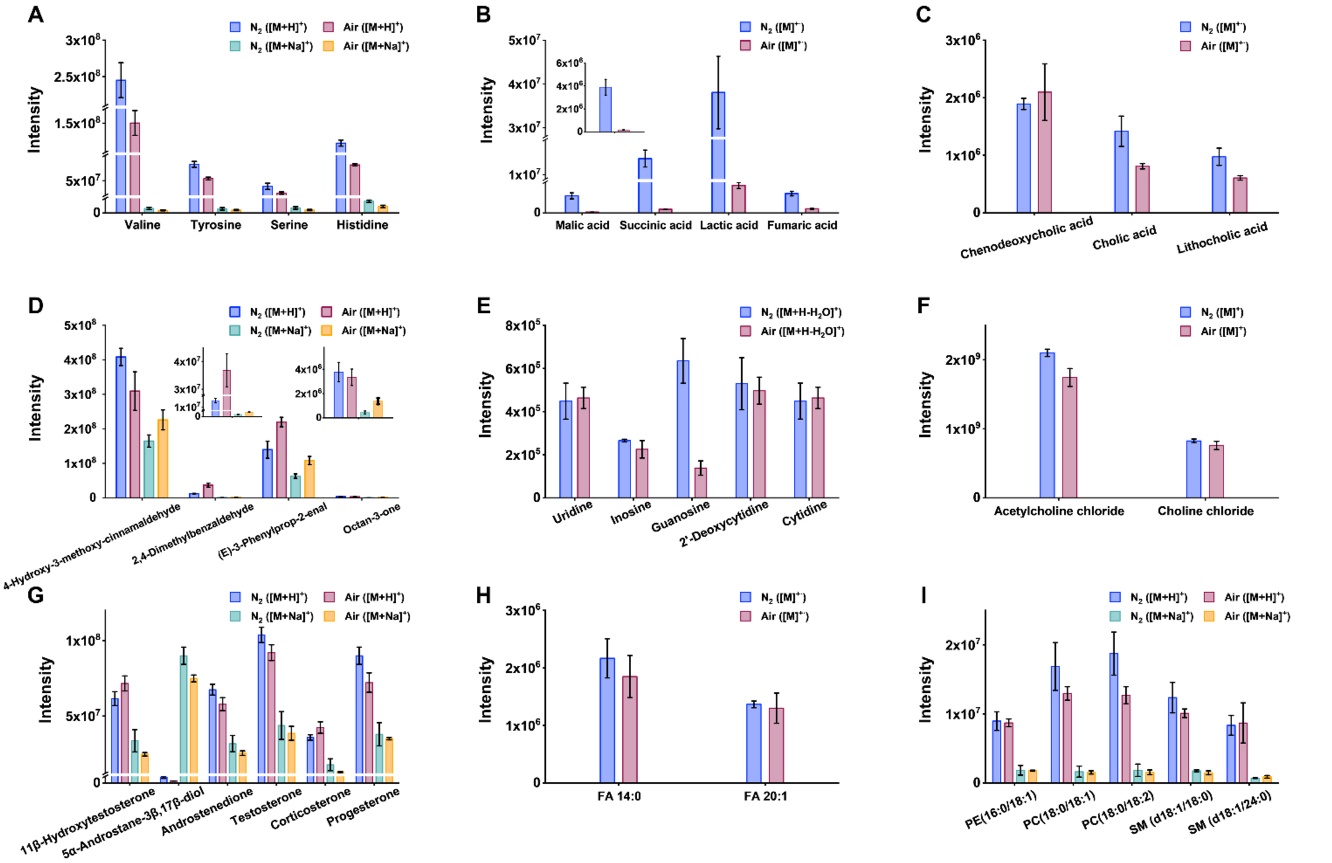

Figure S3. Effect of plasma gases (N_2, air) on the intensity of 9 classes of model compounds with different polarities: (A) AAs, (B) TCAs, (C) BAs, (D) aldehydes and ketones, (E) nucleosides, (F) ionic species, (G) sterols, (H) FAs and (I) lipids.

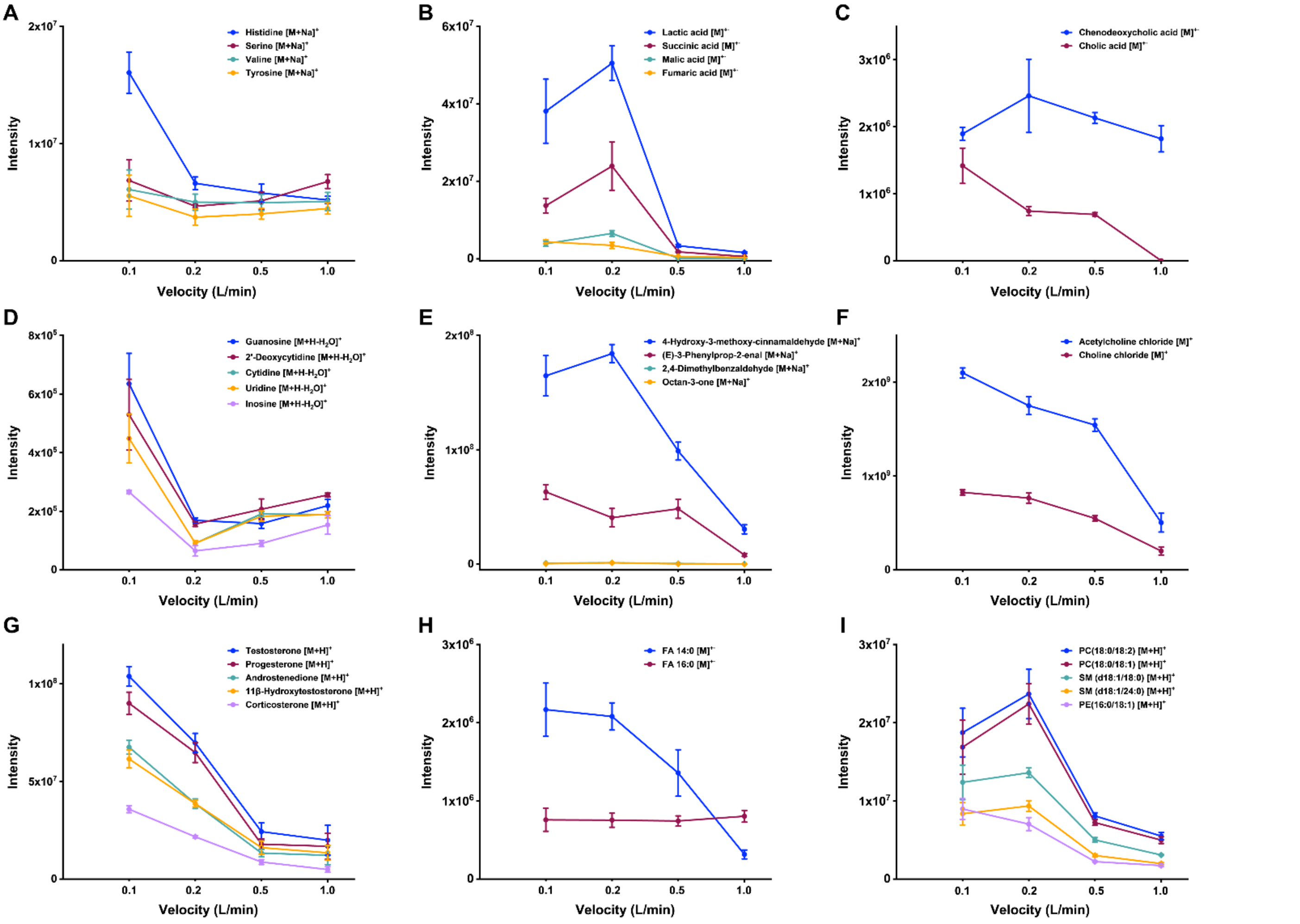

Figure S4. Effect of velocity of N_2 (0.1 L/min, 0.2 L/min, 0.5 L/min 1.0 L/min) on the intensity of 9 classes of model compounds with different polarities: (A) AAs, (B) TCAs, (C) BAs, (D) nucleosides, (E) aldehydes and ketones, (F) ionic species, (G) sterols, (H) FAs and (I) lipids.

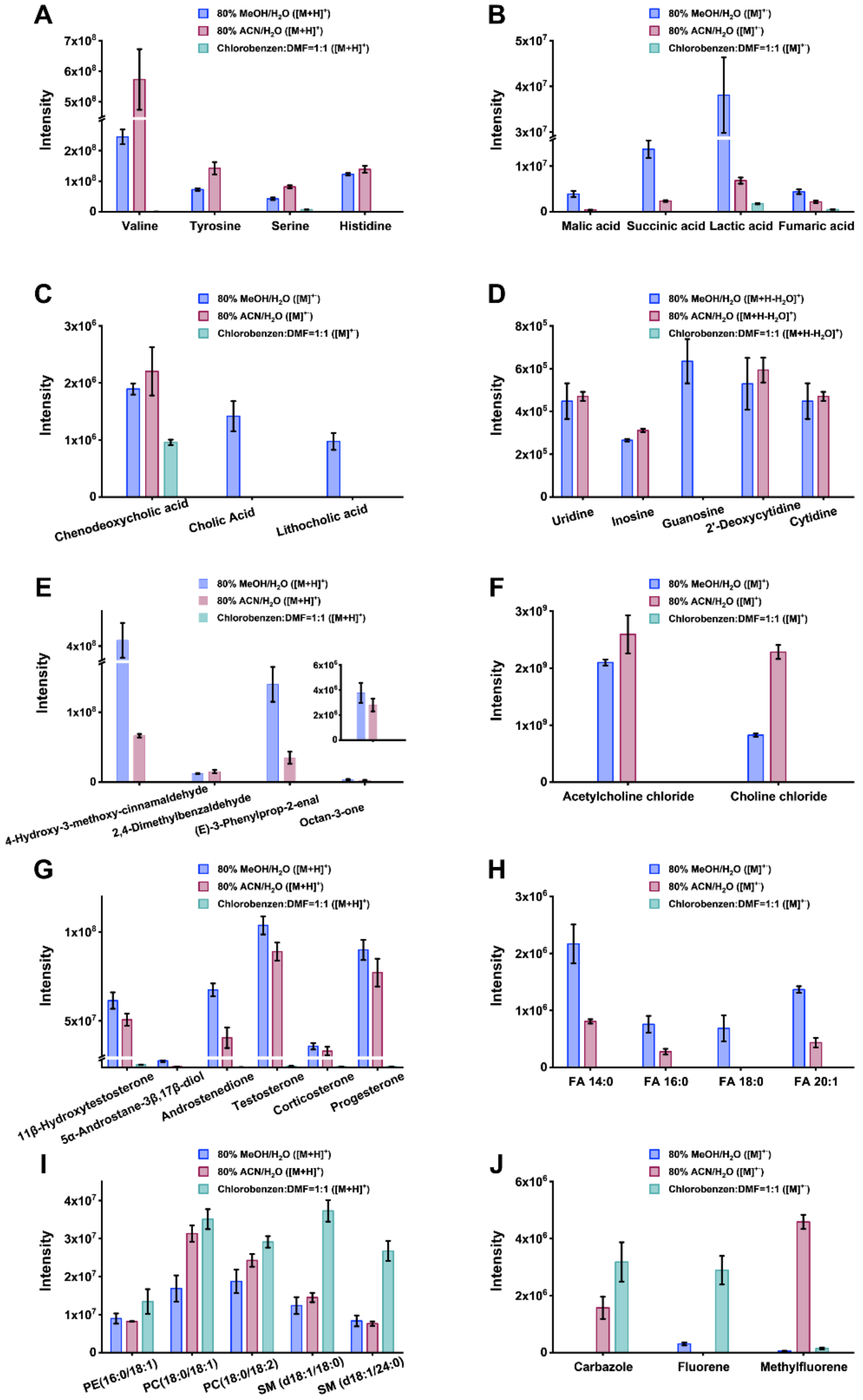

Figure S5. Effect of assistant solvents (80% MeOH/H_2O, 80% ACN/H_2O, chlorobenzene: DMF = 1:1) on the intensity of 10 classes of model compounds with different polarities: (A) AAs, (B) TCAs, (C) BAs, (D) nucleosides, (E) aldehydes and ketones, (F) ionic species, (G) sterols, (H) FAs, (I) lipids and (J) PAHs.

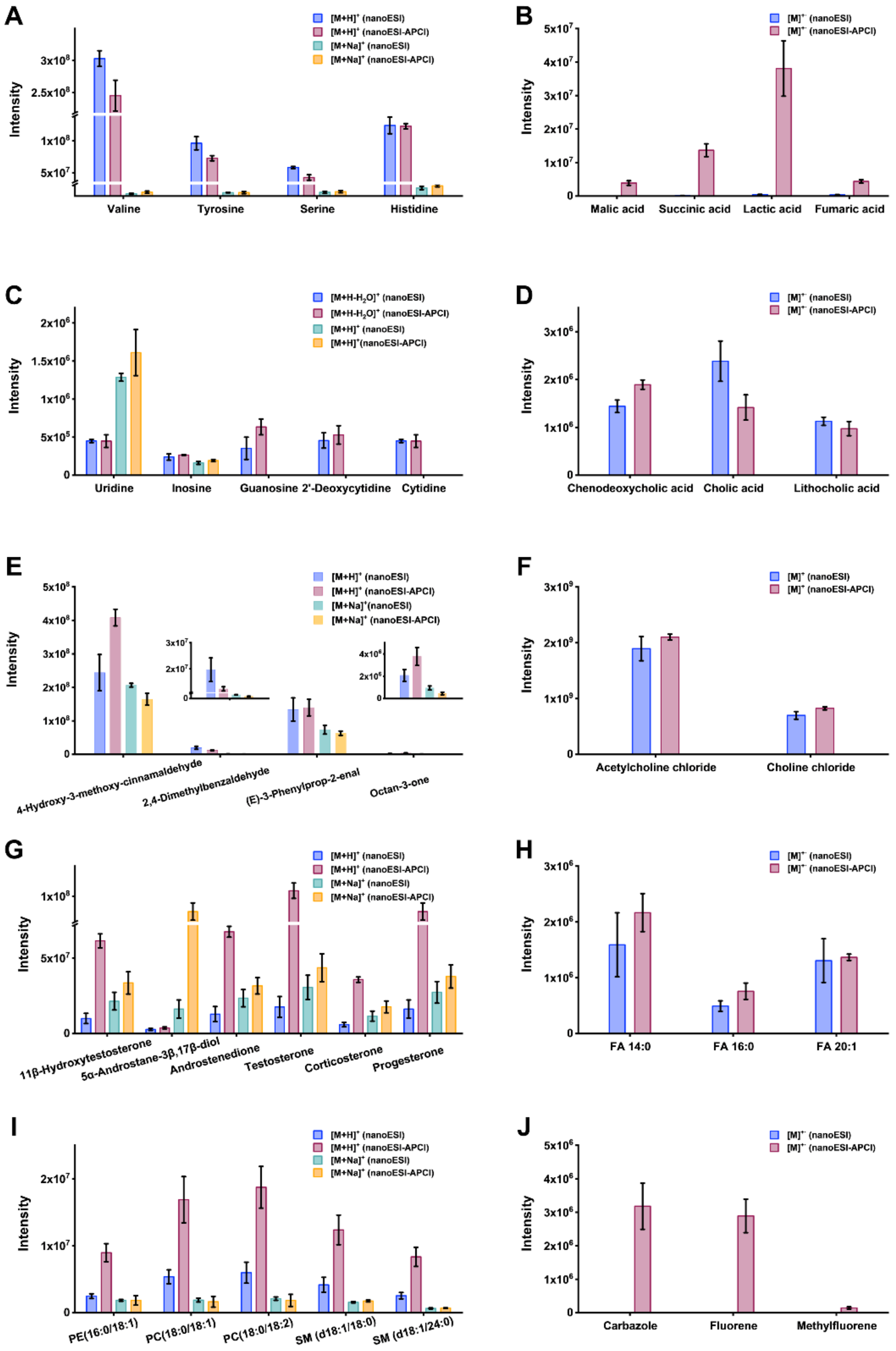

Figure S6. Analysis of 10 classes of model compounds with different polarities in nanoESI mode and nanoESI-APCI mode: (A) AAs, (B) TCAs, (C) nucleosides, (D) BAs, (E) aldehydes and ketones, (F) ionic species, (G) sterols, (H) FAs, (I) lipids and (J) PAHs

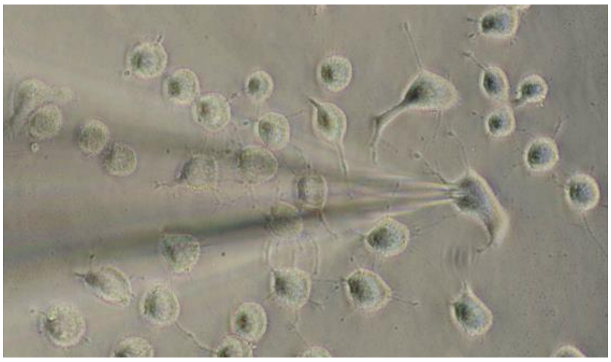

Figure S7. Photograph of an actual sampling of a single MCF7 cell.

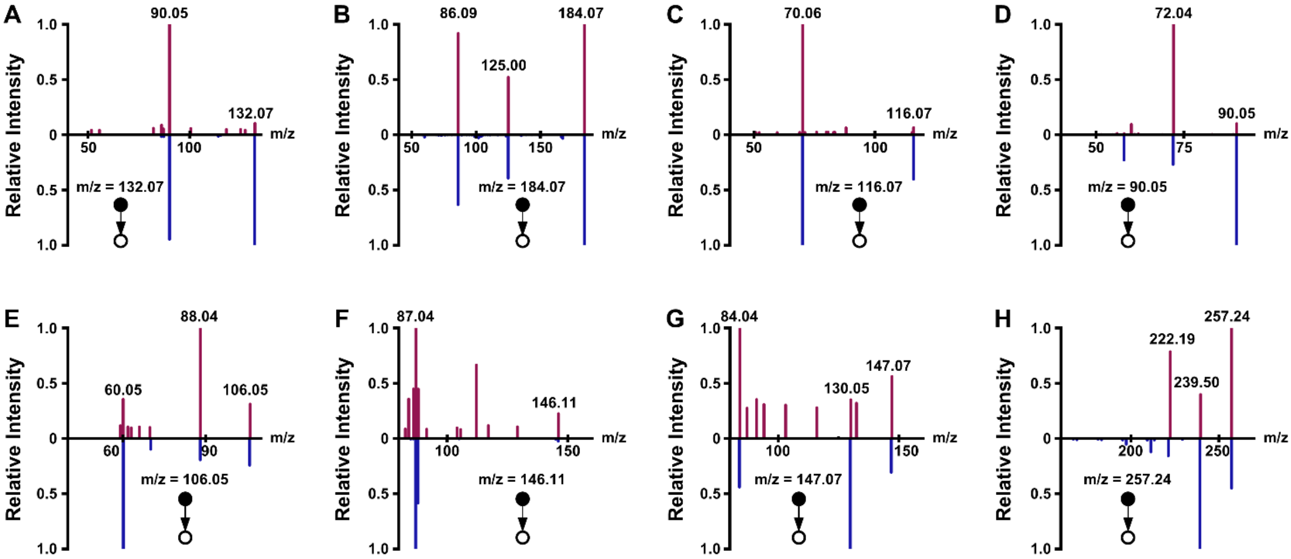

Figure S8. MS2 spectra of (A) creatine, (B) phosphorylcholine, (C) proline, (D) alanine, (E) serine, (F) acetylcholine, (G) glutamine and (H) palmitic acid obtained from a single MCF7 cell. Up panel: measurement, down panel: reference.

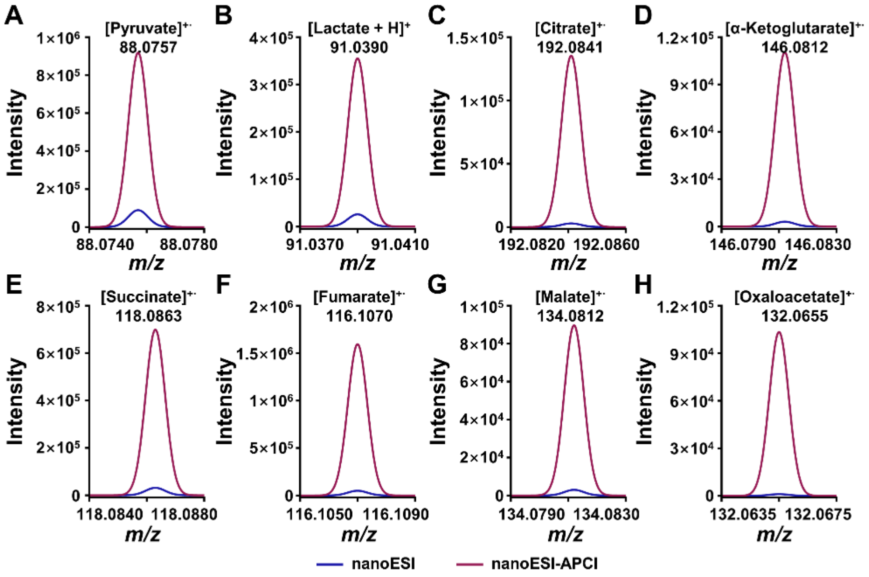

Figure S9. Intensity comparisons of 8 key metabolites in nanoESI mode and nanoESI-APCI mode. (A) Pyruvate, (B) lactate, (C) citrate, (D) α-ketoglutarate, (E) succinate, (F) fumarate, (G) malate, (H) oxaloacetate.

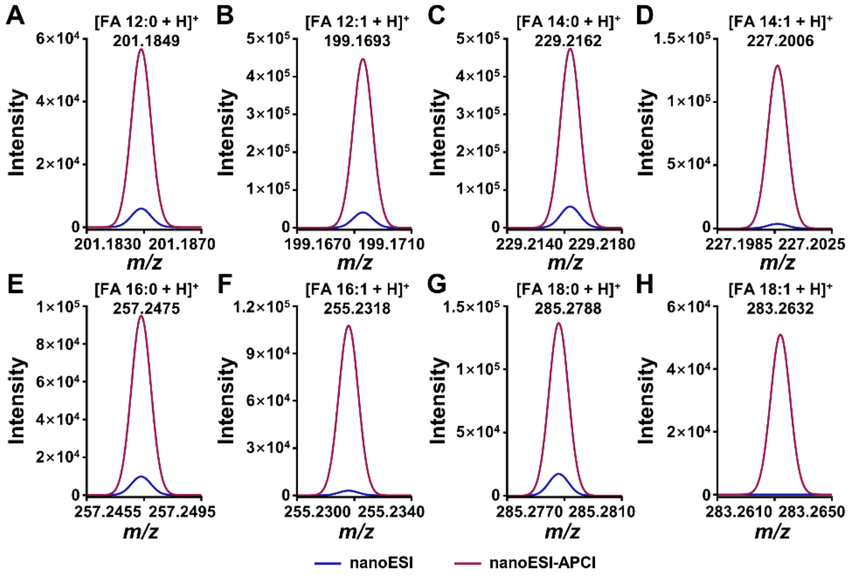

Figure S10. Intensity comparisons of 8 non-polar metabolites in nanoESI mode and nanoESI-APCI mode. (A) FA 12:0, (B) FA 12:1, (C) FA 14:0, (D) FA 14:1, (E) FA 16:0, (F) FA 16:1, (G) FA 18:0, (H) FA 18:1

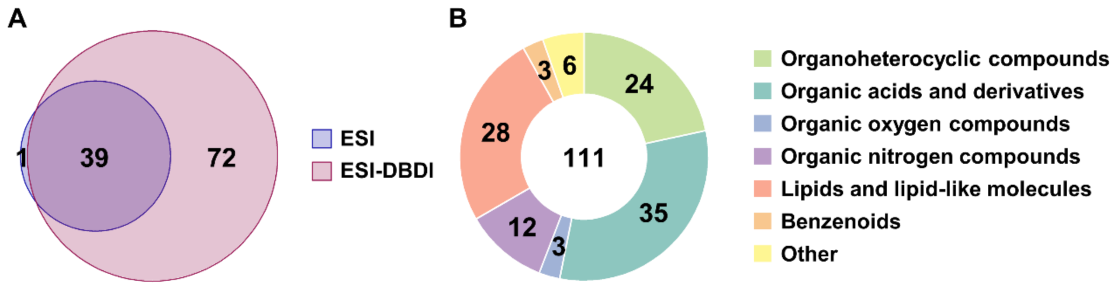

Figure S11. Metabolite analysis in a single PANC-1 cell analyzed by the hybrid ESI-DBDI source proposed by Zenobi's group. (A) Venn diagram of detected metabolite numbers in ESI-only mode and ESI-DBDI mode. (B) Classifications of detected metabolites in ESI-DBDI mode. The original data were obtained from the Supporting Information of Ref.[5].

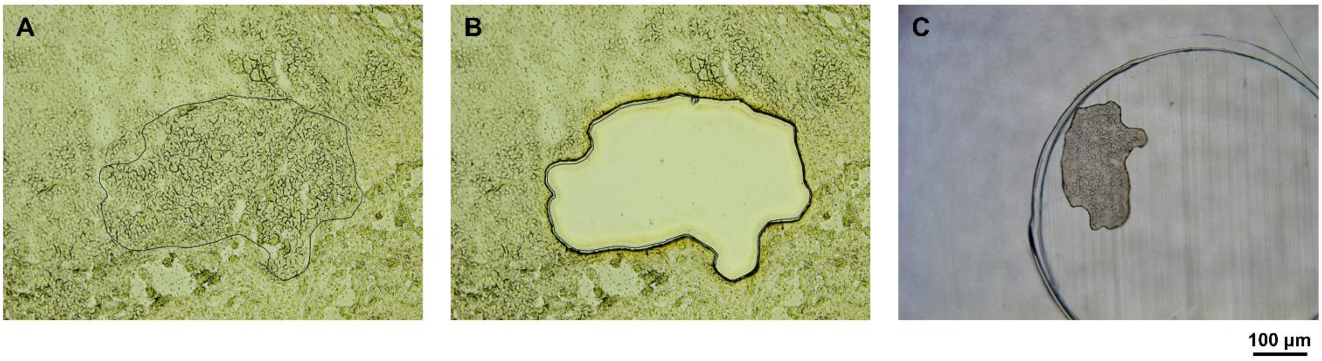

Figure S12. The laser 'cut and drop' sampling process. (A) Image of target tissue microregion in HCC tissue section obtained by LCM. (B) Image of HCC tissue section after microdissection. (C) Image of target tissue microregion collected in the collection tube.

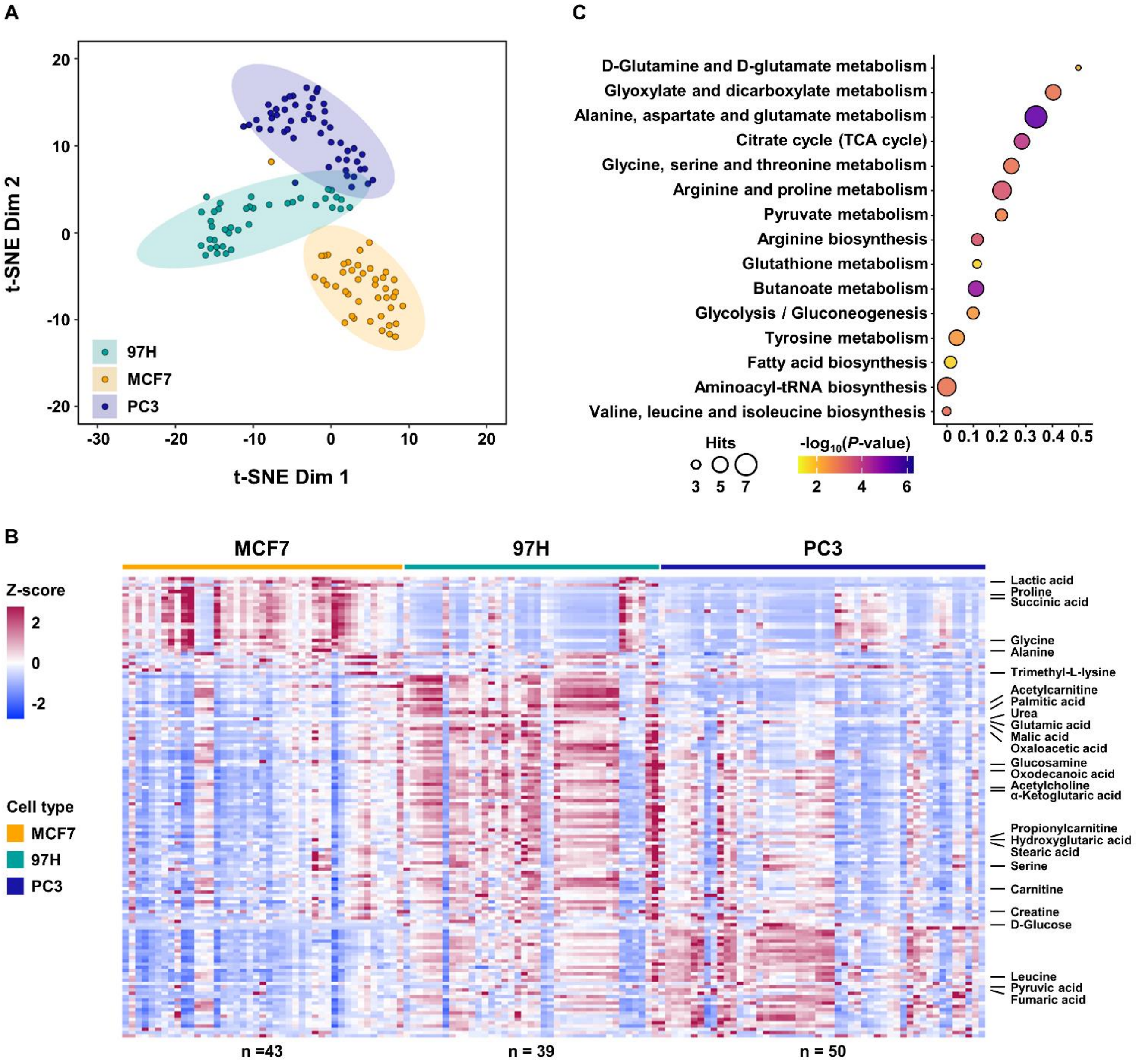

Figure S13. Discrimination results of cancer cell types. (A) The t-SNE plot with 95% confidence ellipses of three types of cancer cell lines including 43 MCF7 cells, 39 97H cells and 50 PC3 cells. (B) Heatmap of significantly changed metabolites with $p < 0.05$ for the discrimination of cancer cell types. Color indicates *z*-scores of metabolites. (C) KEGG metabolic pathways differentially regulated in three different cancer cell types. Each circle was colored by the $-\log_{10}(p\text{-value})$ and the size was correlated to matched metabolites

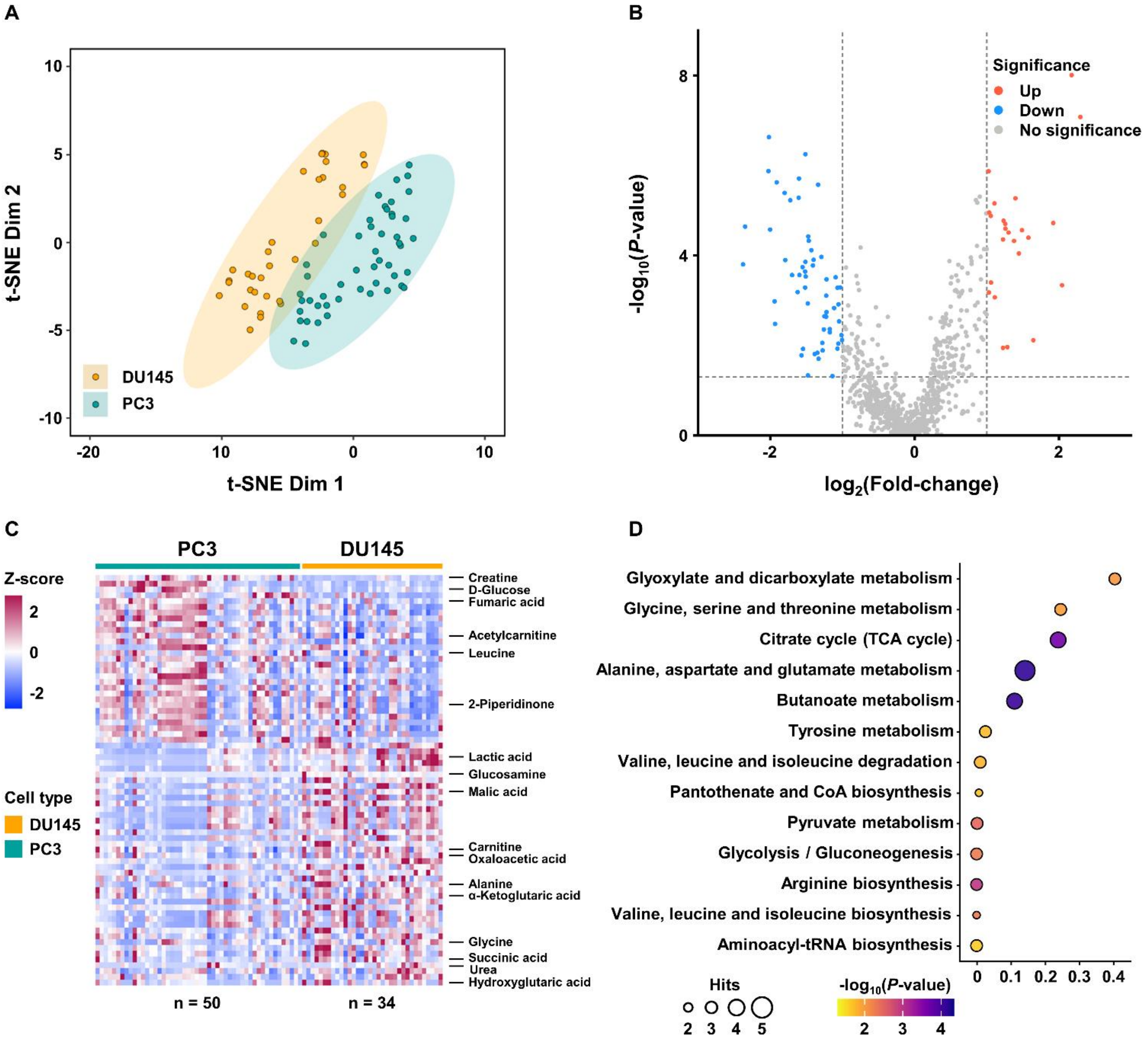

Figure S14. Discrimination results of PCa cell subtypes. (A) The t-SNE plot with 95% confidence ellipses of two subtypes of prostate cancer including 50 PC3 cells and 34 DU145 cells. (B) Volcano plot showing correlations between *p*-value and fold-change for all metabolites in PC3 and DU145 cells. Characteristic metabolites are highlighted in red (upregulated) and blue (downregulated). (C) Heatmap of significantly changed metabolites with $p < 0.05$ for the discrimination of PC3 and DU145. Color indicates *z*-scores of metabolites. (D) KEGG metabolic pathways differentially regulated in two different PCa cell subtypes. Each circle was colored by the $-\log_{10}(p\text{-value})$ and the size was correlated to the number of matched metabolites.

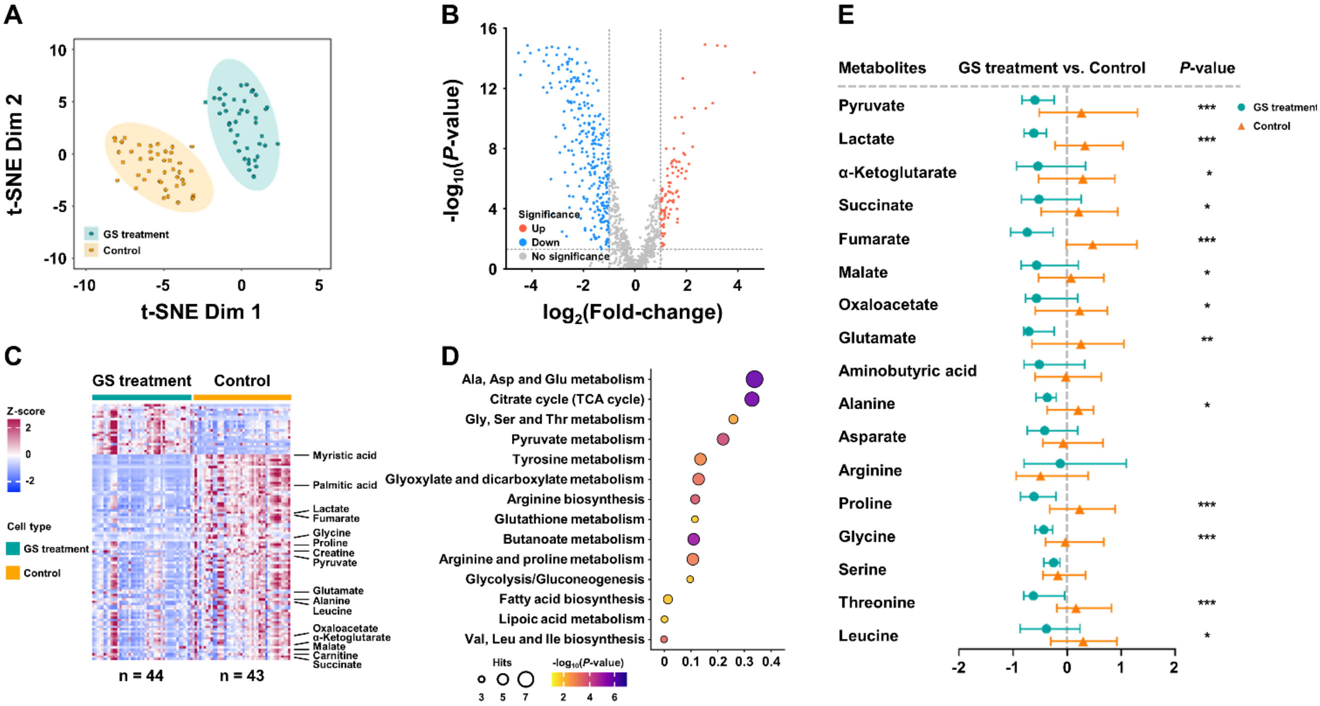

Figure S15. The influence of GS treatment on metabolites of MCF7 cells. (A) The t-SNE plot with 95% confidence ellipses of 44 GS-treated cells and 43 MCF7 cells. (B) Volcano plot showing correlations between *p*-value and fold-change for all metabolites in GS treatment and control groups. Characteristic metabolites are highlighted in red (upregulated) and blue (downregulated). (C) Heatmap of significantly changed metabolites with $p < 0.05$ for the discrimination of GS treatment and control groups. Color indicates *z*-scores of metabolites. (D) KEGG metabolic pathways differentially regulated in GS-treated cells compared to MCF7 cells. Each circle was colored by the $-\log_{10}(p\text{-value})$ and the size was correlated to the number of matched metabolites. (E) *Z*-score plot of 17 representative metabolites with differential abundance in GS treatment and control groups. Data are presented as median with interquartile range and points are colored by assigned cell type. *: $0.001 < p < 0.05$, **: $0.001 < p < 0.0001$, ***: $p < 0.0001$.

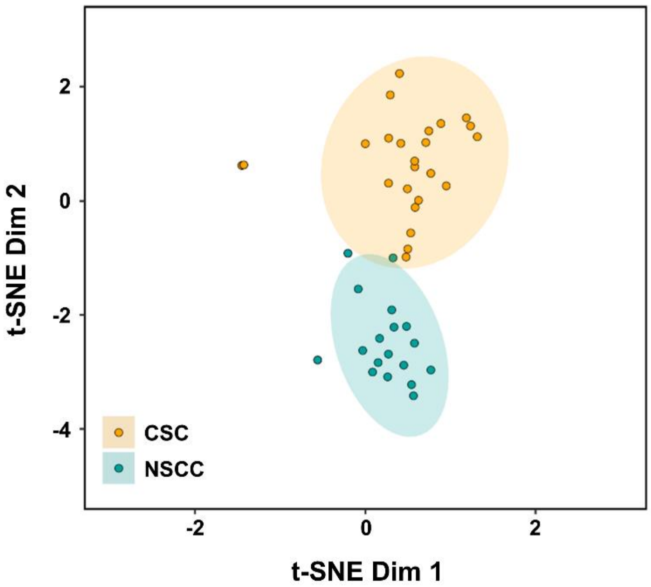

Figure S16. The outcome of t-SNE cluster analysis with 95% confidence ellipses of metabolites with statistically significant differences ($p < 0.05$) between 24 CSCs and 18 NSCCs.

Supporting Tables

Table S1. Detailed information of 10 classes of model compounds.

Classes	Mode compounds	Molecular weight	LogP	Adduct ions	
				nanoESI mode	nanoESI-APCI mode
AAs	Valine	117.0657	-2.26	$[M+H]^+$, $[M+Na]^+$	$[M+H]^+$, $[M+Na]^+$
	Tyrosine	181.0738	-2.26	$[M+H]^+$, $[M+Na]^+$	$[M+H]^+$, $[M+Na]^+$
	Serine	105.0426	-3.07	$[M+H]^+$, $[M+Na]^+$	$[M+H]^+$, $[M+Na]^+$
	Histidine	155.0694	-3.32	$[M+H]^+$, $[M+Na]^+$	$[M+H]^+$, $[M+Na]^+$
TCAs	Malic acid	134.0212	-1.26	-	$[M]^{+\bullet}$
	Succinic acid	118.0266	-0.59	$[M]^{+\bullet}$	$[M]^{+\bullet}$
	Lactic acid	90.0317	-0.72	$[M]^{+\bullet}$	$[M]^{+\bullet}$
	Fumaric acid	116.0120	0.46	$[M]^{+\bullet}$	$[M]^{+\bullet}$
BAs	Chenodeocycholic acid	392.2923	4.15	$[M]^{+\bullet}$	$[M]^{+\bullet}$
	Cholic acid	408.2875	2.02	$[M]^{+\bullet}$	$[M]^{+\bullet}$
	Lithocholic acid	376.2977	6.30	$[M]^{+\bullet}$	$[M]^{+\bullet}$
Nucleosides	Uridine	244.0695	-1.98	$[M+H-H_2O]^+$, $[M+H]^+$	$[M+H-H_2O]^+$, $[M+H]^+$
	Inosine	268.0808	-2.10	$[M+H-H_2O]^+$, $[M+H]^+$	$[M+H-H_2O]^+$, $[M+H]^+$
	Guanosine	283.0917	-1.90	$[M+H-H_2O]^+$	$[M+H-H_2O]^+$
	2'-Deoxycytidine	227.0906	-1.77	$[M+H-H_2O]^+$	$[M+H-H_2O]^+$
	Cytidine	243.0855	-2.51	$[M+H-H_2O]^+$	$[M+H-H_2O]^+$
Aldehydes and ketones	4-Hydroxy-3-methoxy-cinnamaldehyde	178.0630	1.50	$[M+H]^+$, $[M+Na]^+$	$[M+H]^+$, $[M+Na]^+$
	2,4-Dimethylbenzaldehyde	134.0732	2.50	$[M+H]^+$, $[M+Na]^+$	$[M+H]^+$, $[M+Na]^+$
	(E)-3-Phenylprop-2-enal	132.0575	1.90	$[M+H]^+$, $[M+Na]^+$	$[M+H]^+$, $[M+Na]^+$
	Octan-3-one	128.1201	2.30	$[M+H]^+$, $[M+Na]^+$	$[M+H]^+$, $[M+Na]^+$
Ionic species	Acetylcholine chloride	181.6600	-4.20	$[M]^{+\bullet}$	$[M]^{+\bullet}$
	Choline chloride	139.6200	-5.16	$[M]^{+\bullet}$	$[M]^{+\bullet}$

Classes	Mode compounds	Molecular weight	LogP	Adduct ions	
				nanoESI mode	nanoESI-APCI mode
Sterols	11β-Hydroxytestosterone	304.2038	2.20	$[M+H]^+$, $[M+Na]^+$	$[M+H]^+$, $[M+Na]^+$
	5α-Androstane-3β,17β-diol	292.2402	4.20	$[M+H]^+$, $[M+Na]^+$	$[M+H]^+$, $[M+Na]^+$
	Androstenedione	286.1933	2.75	$[M+H]^+$, $[M+Na]^+$	$[M+H]^+$, $[M+Na]^+$
	Testosterone	288.2089	3.32	$[M+H]^+$, $[M+Na]^+$	$[M+H]^+$, $[M+Na]^+$
	Corticosterone	346.2144	1.94	$[M+H]^+$, $[M+Na]^+$	$[M+H]^+$, $[M+Na]^+$
	Progesterone	314.2246	3.87	$[M+H]^+$, $[M+Na]^+$	$[M+H]^+$, $[M+Na]^+$
FAs	FA 14:0	228.2089	6.11	$[M]^{+\bullet}$	$[M]^{+\bullet}$
	FA 16:0	256.2402	7.17	$[M]^{+\bullet}$	$[M]^{+\bullet}$
	FA 18:0	284.2712	8.23	$[M]^{+\bullet}$	$[M]^{+\bullet}$
	FA 20:1	310.2872	7.60	$[M]^{+\bullet}$	$[M]^{+\bullet}$
Lipids	PE (16:0/18:1)	717.5308	10.40	$[M+H]^+$, $[M+Na]^+$	$[M+H]^+$, $[M+Na]^+$
	PC (18:0/18:1)	787.6091	14.70	$[M+H]^+$, $[M+Na]^+$	$[M+H]^+$, $[M+Na]^+$
	PC (18:0/18:2)	785.5934	13.80	$[M+H]^+$, $[M+Na]^+$	$[M+H]^+$, $[M+Na]^+$
	SM (d18:1/18:0)	730.5989	13.40	$[M+H]^+$, $[M+Na]^+$	$[M+H]^+$, $[M+Na]^+$
	SM (d18:1/24:0)	814.6927	16.60	$[M+H]^+$, $[M+Na]^+$	$[M+H]^+$, $[M+Na]^+$
PAHs	Carbazole	167.0735	3.70	-	$[M]^{+\bullet}$
	Fluorene	166.0783	4.20	-	$[M]^{+\bullet}$
	Methylfluorene	180.2500	5.00	-	$[M]^{+\bullet}$

Table S2. LOD and linear range acquired in both modes for different model compounds.

Metabolite	LOD (ng/mL)		Linear Range (ng/mL)			
	nanoESI	nanoESI-APCI	nanoESI	R^2	nanoESI-APCI	R^2
Histidine	0.1	0.01	1-1000	0.9915	0.1-1000	0.9917
Guanosine	0.1	0.01				
4-Hydroxy-3-methoxy-cinnamaldehyde	0.1	0.01	5-1000	0.9948	0.05-500	0.9955
Corticosterone	0.05	0.01	0.2-500	0.9965	0.05-500	0.9944
FA 14:0	0.5	0.1				
PC 18:0/18:1	0.5	0.25	1-5000	0.9922	0.5-5000	0.9966
Carbazole	0.5	0.25				

Table S3. Assigned metabolites from a single cell in nanoESI and nanoESI-APCI modes.

No.	Metabolite name	Molecular formula	Exact mass	Obsmz	Theomz	Adduct	Error, ppm	nanoESI	nanoESI-APCI	LC-MS
1	Pyrrolidine	C_4H_9N	71.0735	72.0808	72.0808	M+H	0.01	√	√	*
2	Acrylic acid	$C_3H_4O_2$	72.0211	73.0284	73.0284	M+H	0.4	√	√	*
3	3-Aminopropionaldehyde	C_3H_7NO	73.0528	74.06	74.0601	M+H	0.86	√	√	*
				91.0865	91.0866	$M+NH_4$	0.7	-	√	
				96.0419	96.042	M+Na	0.66	√	√	
4	Methylguanidine	$C_2H_7N_3$	73.064	74.0712	74.0713	M+H	1.31	-	√	
5	Butylamine	$C_4H_{11}N$	73.0891	74.0964	74.0964	M+H	0.67	√	√	*
6	Lactaldehyde	$C_3H_6O_2$	74.0368	75.044	75.0441	M+H	1.06	√	√	*
				92.0705	92.0706	$M+NH_4$	0.86	-	√	
				97.0259	97.026	M+Na	0.82	-	√	
7	Glycine	$C_2H_5NO_2$	75.032	76.0393	76.0393	M+H	0.37	√	√	*
				93.0658	93.0658	$M+NH_4$	0.31	-	√	
				98.0212	98.0212	M+Na	0.29	-	√	
8	Urea	CH_4N_2O	60.0324	83.0215	83.0216	M+Na	0.76	√	√	*
9	Pentadienal	C_5H_6O	82.0419	83.0491	83.0492	M+H	0.78	√	√	
10	Dimethyl peroxide	$C_2H_6O_2$	62.0368	85.0259	85.026	M+Na	0.93	√	√	*
11	Pyrrolidinone	C_4H_7NO	85.0528	86.06	86.0601	M+H	0.74	-	√	*
				108.042	108.042	M+Na	0.33	-	√	

No.	Metabolite name	Molecular formula	Exact mass	Obsmz	Theomz	Adduct	Error, ppm	nanoESI	nanoESI-APCI	LC-MS
12	4-Hydroxy-2-butenoic acid γ-lactone	$C_4H_4O_2$	84.0211	85.0284	85.0284	M+H	0.35	√	√	
13	Imidazolone	$C_3H_4N_2O$	84.0324	85.0396	85.0397	M+H	0.74	-	√	*
				102.066	102.0662	$M+NH_4$	1.59	-	√	
				107.0215	107.0216	M+Na	0.59	-	√	
14	γ-Butyrolactone	$C_4H_6O_2$	86.0368	87.044	87.0441	M+H	0.91	√	√	*
				109.0259	109.026	M+Na	0.73	√	√	
15	Prenol	$C_5H_{10}O$	86.0732	87.0804	87.0805	M+H	0.75	√	√	
16	Pyruvic acid	$C_3H_4O_3$	88.016	88.0756	88.0757	M+·	1.29	√	√	
17	Aminobutyraldehyde	C_4H_9NO	87.0684	105.1022	105.1022	$M+NH_4$	0.13	-	√	
				110.0576	110.0576	M+Na	0.13	√	√	
18	3-Methyl-1-butylamine	$C_5H_{13}N$	87.1048	88.112	88.1121	M+H	1.13	√	√	
19	Butyric acid	$C_4H_8O_2$	88.0524	89.0596	89.0597	M+H	1.45	√	√	*
				106.0862	106.0862	$M+NH_4$	0.28	√	√	
				111.0416	111.0416	M+Na	0.27	√	√	
20	Methyl-1-butanol	$C_5H_{12}O$	88.0888	89.096	89.0961	M+H	1.29	√	√	
21	Alanine	$C_3H_7NO_2$	89.0477	90.0549	90.055	M+H	0.87	√	√	*
				107.0814	107.0815	$M+NH_4$	0.73	-	√	
				112.0368	112.0369	M+Na	0.7	√	√	
22	Dimethylethanolamine	$C_4H_{11}NO$	89.0841	90.0913	90.0914	M+H	0.71	√	√	

No.	Metabolite name	Molecular formula	Exact mass	Obsmz	Theomz	Adduct	Error, ppm	nanoESI	nanoESI-APCI	LC-MS
23	Lactic acid	$C_3H_6O_3$	90.0317	91.0389	91.039	M+H	1.03	√	√	*
				108.0654	108.0655	$M+NH_4$	0.87	-	√	
				113.0208	113.0209	M+Na	0.83	√	√	
24	Butanediol	$C_4H_{10}O_2$	90.0681	91.0753	91.0754	M+H	0.87	√	√	*
				108.1017	108.1019	$M+NH_4$	1.66	√	√	
				113.0571	113.0573	M+Na	1.59	√	√	
25	Glyoxylic acid	$C_2H_2O_3$	74.0004	92.0341	92.0342	$M+NH_4$	1.02	-	√	
26	Glycerol	$C_3H_8O_3$	92.0473	93.0545	93.0546	M+H	1.55	√	√	*
				110.081	110.0811	$M+NH_4$	1.31	√	√	
				115.0365	115.0365	M+Na	0.38	√	√	*
27	Dimethyl sulfone	$C_2H_6O_2S$	94.0089	95.0161	95.0162	M+H	0.53	-	√	
				112.0426	112.0427	$M+NH_4$	0.45	-	√	*
				116.9979	116.9981	M+Na	1.28	-	√	
28	2-Methyl-2-cyclopenten-1-one	C_6H_8O	96.0575	97.0647	97.0648	M+H	1.18	√	√	
29	Phosphate	H_3O_4P	97.9769	98.9841	98.9842	M+H	0.96	√	√	*
				116.0106	116.0107	$M+NH_4$	0.82	√	√	
				120.966	120.9661	M+Na	0.79	√	√	
30	Furanmethanol	$C_5H_6O_2$	98.0368	99.044	99.0441	M+H	0.8	√	√	*
31	2-Methyl-4-pentenal	$C_6H_{10}O$	98.0732	99.0804	99.0805	M+H	0.66	√	√	
32	Succinic anhydride	$C_4H_4O_3$	100.016	101.0232	101.0233	M+H	1.43	√	√	*
33	Hexylamine	$C_6H_{15}N$	101.1204	102.1276	102.1277	M+H	1.46	√	√	

No.	Metabolite name	Molecular formula	Exact mass	Obsmz	Theomz	Adduct	Error, ppm	nanoESI	nanoESI-APCI	LC-MS
34	Methylpentanal	$C_6H_{12}O$	100.0888	101.096	101.0961	M+H	1.14	√	√	
				118.1225	118.1226	$M+NH_4$	0.97	-	√	*
35	3-Aminodihydro-2(3H)-furanone	$C_4H_7NO_2$	101.0477	102.0549	102.055	M+H	0.77	√	√	*
				124.0369	124.0369	M+Na	0.17	-	√	
36	Ketobutyric acid	$C_4H_6O_3$	102.0317	103.0389	103.039	M+H	0.91	√	√	*
				125.0209	125.0209	M+Na	0.05	√	√	
37	Pentanoic acid	$C_5H_{10}O_2$	102.0681	103.0752	103.0754	M+H	1.74	√	√	*
38	2-Ethoxybutane	$C_6H_{14}O$	102.1045	103.1116	103.1118	M+H	1.6	√	√	
39	Aminobutanoic acid	$C_4H_9NO_2$	103.0632	104.0705	104.0705	M+H	0	√	√	*
40	Hydroxybutyric acid	$C_4H_8O_3$	104.0473	105.0546	105.0546	M+H	0.42	√	√	*
				122.0811	122.0811	$M+NH_4$	0.36	-	√	*
				127.0365	127.0365	M+Na	0.35	√	√	
41	Diaminopropionic acid	$C_3H_8N_2O_2$	104.0586	105.0658	105.0659	M+H	0.74	√	√	
				127.0477	127.0478	M+Na	0.61	-	√	
42	Serine	$C_3H_7NO_3$	105.0426	106.0498	106.0499	M+H	0.88	-	√	*
				123.0763	123.0764	$M+NH_4$	0.76	-	√	
				128.0317	128.0318	M+Na	0.73	-	√	
43	3-Ethylpyridine	C_7H_9N	107.0735	108.0806	108.0808	M+H	1.84	√	√	*
44	5-Methyl-2-furancarboxaldehyde	$C_6H_6O_2$	110.0363	111.0441	111.0441	M+H	0.19	√	√	*
45	3-Hydroxy-4-aminopyridine	$C_5H_6N_2O$	110.048	111.0551	111.0553	M+H	1.92	-	√	

No.	Metabolite name	Molecular formula	Exact mass	Obsmz	Theomz	Adduct	Error, ppm	nanoESI	nanoESI-APCI	LC-MS
46	3-Methyl-2-cyclohexen-1-one	$C_7H_{10}O$	110.0732	111.0803	111.0805	M+H	1.48	√	√	
47	Acetyl-2,3-dihydro-1H-pyrrole	C_6H_9NO	111.0684	112.0756	112.0757	M+H	1.02	√	√	
				129.1021	129.1022	$M+NH_4$	0.88	√	√	*
48	1,2-Cyclohexanedione	$C_6H_8O_2$	112.0524	113.0595	113.0597	M+H	2.03	√	√	*
				135.0415	135.0416	M+Na	0.96	√	√	
49	Heptenal	$C_7H_{12}O$	112.0888	113.096	113.0961	M+H	1.02	√	√	
				130.1226	130.1226	$M+NH_4$	0.12	√	√	
50	Pyrroline-5-carboxylic acid	$C_5H_7NO_2$	113.0477	114.0548	114.055	M+H	1.56	√	√	
51	Creatinine	$C_4H_7N_3O$	113.0589	114.0661	114.0662	M+H	0.98	-	√	*
				136.0479	136.0481	M+Na	1.56	√	√	
52	Cis-Acetylacrylate	$C_5H_6O_3$	114.0312	115.0392	115.039	M+H	1.79	√	√	*
53	Dihydrouracil	$C_4H_6N_2O_2$	114.0429	115.0501	115.0502	M+H	1.11	-	√	*
54	Hexanolactone	$C_6H_{10}O_2$	114.0681	115.0753	115.0754	M+H	0.69	√	√	*
				137.0573	137.0573	M+Na	0.15	√	√	
55	Hepten-1-ol	$C_7H_{14}O$	114.1045	115.1116	115.1118	M+H	1.43	-	√	
				132.1382	132.1383	$M+NH_4$	0.49	-	√	
56	Proline	$C_5H_9NO_2$	115.0633	116.0705	116.0706	M+H	1.11	√	√	*
				138.0525	138.0525	M+Na	0.21	√	√	*
57	Fumaric acid	$C_4H_4O_4$	116.011	116.1069	116.107	M+·	0.98	√	√	
58	Ketoisovaleric acid	$C_5H_8O_3$	116.0473	117.0545	117.0546	M+H	1.23	√	√	
				139.0365	139.0365	M+Na	0.32	√	√	

No.	Metabolite name	Molecular formula	Exact mass	Obsmz	Theomz	Adduct	Error, ppm	nanoESI	nanoESI-APCI	LC-MS
59	N-(3-Methylbutyl)acetamide	$C_6H_{13}NO$	115.0997	133.1335	133.1335	$M+NH_4$	0.11	-	√	
60	Nitroso-3-hydroxypyrrolidine	$C_4H_8N_2O_2$	116.0586	117.0657	117.0659	M+H	1.52	√	√	*
61	2-Methyl-1-methylthio-2-butene	$C_6H_{12}S$	116.066	117.0738	117.0733	M+H	4.52	√	√	
62	Caproic acid	$C_6H_{12}O_2$	116.0837	117.0909	117.091	M+H	1.11	√	√	
				134.1175	134.1175	$M+NH_4$	0.22	√	√	
				139.0729	139.0729	M+Na	0.21	√	√	
63	Acetylglycine	$C_4H_7NO_3$	117.0426	118.0497	118.0499	M+H	1.64	√	√	*
				135.0763	135.0764	$M+NH_4$	0.69	-	√	
				140.0318	140.0318	M+Na	0.05	-	√	
64	Guanidoacetic acid	$C_3H_7N_3O_2$	117.0538	118.0609	118.0611	M+H	1.92	-	√	*
65	Succinic acid	$C_4H_6O_4$	118.0266	118.0861	118.0863	M+·	1.51	√	√	
66	Diaminobutyric acid	$C_4H_{10}N_2O_2$	118.0742	119.0815	119.0815	M+H	0.23	-	√	*
67	Methylmalonic acid	$C_4H_6O_4$	118.0266	119.0338	119.0339	M+H	0.91	-	√	
				141.0158	141.0158	M+Na	0.06	-	√	
68	2-Methyl-3-hydroxybutyric acid	$C_5H_{10}O_3$	118.063	119.0702	119.0703	M+H	0.79	√	√	*
				136.0969	136.0968	$M+NH_4$	0.78	√	√	
				141.0521	141.0522	M+Na	0.67	√	√	
69	Threonine	$C_4H_9NO_3$	119.0582	120.0654	120.0655	M+H	1.19	√	√	*
				142.0475	142.0474	M+Na	0.4	√	√	
70	Vinylphenol	C_8H_8O	120.0579	121.0647	121.0652	M+H	4.13	√	√	*
71	Phenylethylamine	$C_8H_{11}N$	121.0891	122.0963	122.0964	M+H	1.22	√	√	*

No.	Metabolite name	Molecular formula	Exact mass	Obsmz	Theomz	Adduct	Error, ppm	nanoESI	nanoESI-APCI	LC-MS
72	Hydroxybenzaldehyde	$C_7H_6O_2$	122.0368	123.0446	123.0441	M+H	4.23	√	√	*
73	Phosphoethanolamine	$C_2H_8NO_4P$	141.0191	124.016	124.0164	M+H-H_2O	3.18	-	√	
74	Anisidine	C_7H_9NO	123.0684	124.0757	124.0757	M+H	0.11	√	√	
75	2,3-Dimethyl-2-cyclohexen-1-one	$C_8H_{12}O$	124.0888	125.0961	125.0961	M+H	0.12	√	√	
76	Dimethylglycine	$C_4H_9NO_2$	103.0633	126.0525	126.0525	M+Na	0.23	√	√	*
77	Methylcytosine	$C_5H_7N_3O$	125.0589	126.0661	126.0662	M+H	0.89	-	√	*
78	2-Amino-3-methylenehexanoic acid	$C_7H_{13}NO_2$	143.0946	126.0913	126.0919	M+H-H_2O	4.99	√	√	
				161.1285	161.1284	M+NH_4	0.44	-	√	
79	5-Aminoimidazole-4-carboxamide	$C_4H_6N_4O$	126.0542	127.0613	127.0615	M+H	1.27	-	√	
80	Hydroxycyclohexylcarboxylic acid	$C_7H_{12}O_3$	144.0786	127.0753	127.0758	M+H-H_2O	3.93	√	√	*
81	Octadien-3-ol	$C_8H_{14}O$	126.1045	127.1117	127.1118	M+H	0.51	√	√	*
				144.1383	144.1383	M+NH_4	0.24	√	√	
				149.0934	149.0937	M+Na	1.78	√	√	
82	Dihydrothymine	$C_5H_8N_2O_2$	128.0586	129.0657	129.0659	M+H	1.38	-	√	*
83	Benzylideneacetone	$C_{10}H_{10}O$	146.0732	129.0698	129.0705	M+H-H_2O	5.15	-	√	
				147.0804	147.0805	M+H	0.44	-	√	
84	Pyroglutamic acid	$C_5H_7NO_3$	129.0426	130.0499	130.0499	M+H	0.05	√	√	*
85	2-Methyl-3-ketovaleric acid	$C_6H_{10}O_3$	130.063	131.0703	131.0703	M+H	0.04	√	√	*
				153.052	153.0522	M+Na	1.27	√	√	
86	Pipecolinic acid	$C_6H_{11}NO_2$	129.0789	130.0863	130.0862	M+H	0.77	√	√	*

No.	Metabolite name	Molecular formula	Exact mass	Obsmz	Theomz	Adduct	Error, ppm	nanoESI	nanoESI-APCI	LC-MS
87	Ethyl isovalerate	$C_7H_{14}O_2$	130.0994	131.1066	131.1067	M+H	0.61	√	√	
				148.1332	148.1332	$M+NH_4$	0.14	√	√	
88	Oxaloacetic acid	$C_4H_4O_5$	132.0059	132.0655	132.0655	M+·	0.33	√	√	
89	Hydroxyproline	$C_5H_9NO_3$	131.0582	154.0474	154.0474	M+Na	0.28	-	√	*
90	Creatine	$C_4H_9N_3O_2$	131.0695	132.0767	132.0768	M+H	0.58	√	√	*
				154.0586	154.0587	M+Na	0.5	√	√	*
91	Leucine	$C_6H_{13}NO_2$	131.0946	132.1019	132.1019	M+H	0.22	√	√	*
				154.0837	154.0838	M+Na	0.84	√	√	
92	Asparagine	$C_4H_8N_2O_3$	132.0535	133.0607	133.0608	M+H	0.69	√	√	*
93	Hydroxyhexanoic acid	C6H12O3	132.0786	133.0859	133.0859	M+H	0.33	-	√	*
				155.0675	155.0678	M+Na	2.22	-	√	*
				150.1125	150.1125	M+NH4	0.04	√	√	*
94	Ornithine	$C_5H_{12}N_2O_2$	132.0899	133.097	133.0972	M+H	1.33	-	√	*
95	Carvone	$C_{10}H_{14}O$	150.1045	133.1012	133.1018	$M+H-H_2O$	4.25	√	√	*
96	Aspartic acid	$C_4H_7NO_4$	133.0375	134.0447	134.0448	M+H	0.8	√	√	*
				156.0267	156.0267	M+Na	0.05	√	√	
97	Malic acid	$C_4H_6O_5$	134.0215	134.0811	134.0812	M+·	0.7	√	√	
98	2-Amino-5-hydroxypentanoic acid	$C_5H_{11}NO_3$	133.0739	156.063	156.0631	M+Na	0.6	-	√	
99	Ethyl 2-mercaptopropionate	$C_5H_{10}O_2S$	134.0402	135.0481	135.0475	M+H	4.81	-	√	
100	(4-Methylphenyl)acetaldehyde	$C_9H_{10}O$	134.0732	135.0804	135.0805	M+H	0.48	√	√	*

No.	Metabolite name	Molecular formula	Exact mass	Obsmz	Theomz	Adduct	Error, ppm	nanoESI	nanoESI-APCI	LC-MS
101	2-Amino-3,4-dihydroxybutanoic acid	$C_4H_9NO_4$	135.0532	136.0605	136.0605	M+H	0.31	-	√	
102	Phenylacetamide	C_8H_9NO	135.0684	136.0758	136.0757	M+H	0.63	√	√	*
103	Methylbenzoic acid	$C_8H_8O_2$	136.0524	137.0598	137.0597	M+H	0.51	√	√	*
				159.0418	159.0416	M+Na	1.07	√	√	
104	Methylnicotinamide	$C_7H_8N_2O$	136.0637	137.071	137.071	M+H	0.27	-	√	*
105	Deoxyerythronic acid	$C_4H_8O_4$	120.0423	138.0761	138.0761	$M+NH_4$	0.3	-	√	
				143.0315	143.0315	M+Na	0.29	-	√	
106	Tyramine	C8H11NO	137.0841	138.0914	138.0914	M+H	0.26	-	√	*
				155.1178	155.1179	M+NH4	0.41	√	√	*
107	Hydroxybenzoic acid	$C_7H_6O_3$	138.0317	139.0392	139.039	M+H	1.48	-	√	
108	3-Acetyl-2,5-dimethylfuran	$C_8H_{10}O_2$	138.0681	139.0754	139.0754	M+H	0.15	√	√	
				161.057	161.0573	M+Na	1.74	√	-	
109	Aminopentanoic acid	C5H11NO2	117.0790	140.0681	140.0682	M+Na	0.56	√	√	*
110	Hexadienyl acetate	$C_8H_{12}O_2$	140.0837	141.091	141.091	M+H	0.21	-	√	
				163.0725	163.0729	M+Na	2.63	-	√	
111	Pyrazin-2-carboxylic acid	$C_5H_4N_2O_2$	124.0273	142.0611	142.0611	$M+NH_4$	0.16	-	√	
112	Hydroxybenzyl alcohol	$C_7H_8O_2$	124.0524	142.0863	142.0863	$M+NH_4$	0.15	√	√	*
113	Methyladipic acid	$C_7H_{12}O_4$	160.0736	143.0704	143.0709	$M+H-H_2O$	3.21	√	√	
				161.0808	161.0809	M+H	0.37	√	√	
				178.1074	178.1074	$M+NH_4$	0.23	√	√	

No.	Metabolite name	Molecular formula	Exact mass	Obsmz	Theomz	Adduct	Error, ppm	nanoESI	nanoESI-APCI	LC-MS
				183.0628	183.0628	M+Na	0.22	√	√	
114	Ala-Ala	$C_6H_{12}N_2O_3$	160.0848	143.0815	143.0821	M+H-H_2O	4.14	-	√	*
115	Octenoic acid	$C_8H_{14}O_2$	142.0988	143.1067	143.1061	M+H	4.19	√	√	*
116	Aminoadipic acid	$C_6H_{11}NO_4$	161.0736	144.0657	144.0659	M+H-H_2O	1.39	-	√	*
117	Imidazoleacetic acid	$C_5H_6N_2O_2$	126.0429	144.0767	144.0767	M+NH_4	0.19	-	√	
118	Proline betaine	$C_7H_{13}NO_2$	143.0946	144.1019	144.1019	M+H	0.2	√	√	*
119	Erythritol	$C_4H_{10}O_4$	122.0579	145.0471	145.0471	M+Na	0.06	√	√	
120	Hydroxyglutamine	$C_5H_{10}N_2O_4$	162.0641	145.0607	145.0614	M+H-H_2O	4.53	-	√	
121	Hydroxylysine	$C_6H_{14}N_2O_3$	162.1004	145.0972	145.0977	M+H-H_2O	3.74	-	√	*
				163.1082	163.1077	M+H	2.81	-	√	
122	Caprylic acid	$C_8H_{16}O_2$	144.115	145.1223	145.1223	M+H	0.21	√	√	
				162.1488	162.1488	M+NH_4	0.18	√	√	
123	Carboxymethylserine	$C_5H_9NO_5$	163.0481	146.0448	146.0454	M+H-H_2O	3.92	-	√	
124	α-Ketoglutaric acid	$C_5H_6O_5$	146.0215	146.0812	146.0812	M+·	0.05	√	√	
125	Guanidinobutanoic acid	$C_5H_{11}N_3O_2$	145.0851	146.0923	146.0924	M+H	0.87	-	√	*
126	Acetylcholine	$C_7H_{15}NO_2$	146.1176	146.1176	146.1172	M+	2.74	√	√	*
127	Spermidine	$C_7H_{19}N_3$	145.1579	146.1652	146.1652	M+H	0.02	√	√	*
				168.1467	168.1471	M+Na	2.36	√	-	
128	Coumaric acid	$C_9H_8O_3$	169.6727	147.0441	147.0442	M+H-H_2O	0.68	√	√	*
129	Glutamine	$C_5H_{10}N_2O_3$	146.0691	147.0765	147.0764	M+H	0.39	√	√	*
				169.0583	169.0583	M+Na	0.25	√	√	*

No.	Metabolite name	Molecular formula	Exact mass	Obsmz	Theomz	Adduct	Error, ppm	nanoESI	nanoESI-APCI	LC-MS
130	Methylglutaric acid	$C_6H_{10}O_4$	146.0579	147.0652	147.0652	M+H	0.06	√	√	*
				164.0917	164.0917	$M+NH_4$	0.05	√	√	
				169.047	169.0471	M+Na	0.64	√	√	
131	Ethyl 2-hydroxy-2-methylbutyrate	$C_7H_{14}O_3$	146.0943	147.1016	147.1016	M+H	0.04	-	√	
				164.1281	164.1281	$M+NH_4$	0.03	-	√	
132	Lysine	$C_6H_{14}N_2O_2$	146.1055	147.1128	147.1128	M+H	0.19	√	√	*
133	Glutamic acid	$C_5H_9NO_4$	147.0532	148.0605	148.0605	M+H	0.29	√	√	*
				170.0423	170.0424	M+Na	0.34	√	√	*
134	2-Amino-6-hydroxyhexanoic acid	$C_6H_{13}NO_3$	147.0895	148.0968	148.0968	M+H	0.29	√	√	*
135	Dihydroxymandelic acid	$C_8H_8O_5$	166.0266	149.0234	149.0239	$M+H-H_2O$	3.41	√	√	*
136	2-Hydroxyglutaric acid	$C_5H_8O_5$	148.0372	149.0449	149.0445	M+H	2.86	-	√	*
				166.0711	166.071	$M+NH_4$	0.76	-	√	
137	Cinnamic acid	$C_9H_8O_2$	148.0522	149.0599	149.0595	M+H	2.68	√	√	*
138	Monoethyl malonic acid	$C_5H_8O_4$	132.0423	150.0761	150.0761	$M+NH_4$	0.27	√	√	
				155.0314	155.0315	M+Na	0.38	√	√	
139	Methamphetamine	$C_{10}H_{15}N$	149.1204	150.1278	150.1277	M+H	0.34	√	√	
140	Methylphenylacetic acid	$C_9H_{10}O_2$	150.0681	151.0757	151.0754	M+H	2.12	-	√	*
141	Geranic acid	$C_{10}H_{16}O_2$	168.1189	151.1118	151.1112	$M+H-H_2O$	3.97	√	√	
142	Dihydroxyvaleric acid	$C_5H_{10}O_4$	134.0579	152.0918	152.0917	$M+NH_4$	0.6	√	√	
				157.0471	157.0471	M+Na	0.06	√	√	

No.	Metabolite name	Molecular formula	Exact mass	Obsmz	Theomz	Adduct	Error, ppm	nanoESI	nanoESI-APCI	LC-MS
143	Dihydroxyacetophenone	$C_8H_8O_3$	152.0473	153.0547	153.0546	M+H	0.37	√	√	
144	2-Isopropyl-1,4-benzenediol	$C_9H_{12}O_2$	152.0837	153.0913	153.091	M+H	1.77	√	√	
				170.1175	170.1175	$M+NH_4$	0.17	√	√	*
145	Decenoic acid	$C_{10}H_{18}O_2$	170.1307	153.1274	153.128	$M+H-H_2O$	3.92	√	√	*
146	Ureidopropionic acid	$C_4H_8N_2O_3$	132.0535	155.0426	155.0427	M+Na	0.59	-	√	*
147	Oxo-nonanoic acid	$C_9H_{14}O_2$	172.1099	155.1066	155.1072	$M+H-H_2O$	3.87	√	√	*
148	Histidine	$C_6H_9N_3O_2$	155.0695	156.0769	156.0768	M+H	0.64	√	√	*
149	Methylisopelletierine	$C_9H_{17}NO$	155.131	156.1383	156.1383	M+H	0.09	√	√	
150	8-Hydroxy-5,6-octadienoic acid	$C_8H_{12}O_3$	156.0786	157.086	157.0859	M+H	0.35	√	√	
				174.1124	174.1124	$M+NH_4$	0.25	√	√	*
151	Nonanedione	$C_9H_{16}O_2$	156.115	157.1223	157.1223	M+H	0.19	√	√	
				174.1488	174.1488	$M+NH_4$	0.17	√	√	
152	Hydroxycitronellol	$C_{10}H_{22}O_2$	174.162	157.1587	157.1593	$M+H-H_2O$	3.69	√	√	
153	3-Methylcrotonylglycine	$C_7H_{11}NO_3$	157.0739	158.0813	158.0812	M+H	0.68	-	√	
154	Homostachydrine	$C_8H_{15}NO_2$	157.1103	158.1176	158.1176	M+H	0	√	√	*
155	4,6-Dioxoheptanoic acid	$C_7H_{10}O_4$	158.0579	159.0655	159.0652	M+H	1.83	√	√	*
156	1-Hydroxy-3-nonanone	$C_9H_{18}O_2$	158.1307	159.138	159.138	M+H	0.13	√	√	
				176.1644	176.1645	$M+NH_4$	0.45	-	√	
157	L-Hypoglycin A	C_7H_{11}NO2	141.079	159.1128	159.1128	$M+NH_4$	0.13	√	√	
158	Methylbutyrylglycine	$C_7H_{13}NO_3$	159.0895	160.0968	160.0968	M+H	0.27	√	√	*
159	Aminooctanoic acid	$C_8H_{17}NO_2$	159.1259	160.1332	160.1332	M+H	0.18	√	√	*

No.	Metabolite name	Molecular formula	Exact mass	Obsmz	Theomz	Adduct	Error, ppm	nanoESI	nanoESI-APCI	LC-MS
				177.1597	177.1597	M+NH4	0.16	√	√	
160	Hydroxyoctanoic acid	$C_8H_{16}O_3$	160.1099	161.1172	161.1172	M+H	0.28	√	√	
				178.1438	178.1437	M+NH4	0.31	-	√	
161	N-Methyl-L-glutamic acid	$C_6H_{11}NO_4$	161.0688	162.0761	162.0761	M+H	0	√	√	*
162	Carnitine	$C_7H_{15}NO_3$	161.1052	162.1124	162.1125	M+H	0.58	√	√	*
163	Hydroxycoumarin	$C_9H_6O_3$	162.0317	163.0389	163.039	M+H	0.58	√	√	*
164	Octanetriol	$C_8H_{18}O_3$	162.1256	163.1328	163.1329	M+H	0.58	√	√	*
				180.1593	180.1594	M+NH4	0.52	√	√	
				185.1145	185.1148	M+Na	1.59	√	√	*
165	Trimethylammoniobutanoic acid	$C_7H_{15}NO_2$	145.1103	163.1439	163.1441	M+NH4	1.1	-	√	*
166	4-ene-Valproic acid	$C_8H_{14}O_2$	142.0994	165.0883	165.0886	M+Na	1.69	√	√	
167	Phenylbutyric acid	$C_{10}H_{12}O_2$	164.0837	165.0913	165.091	M+H	1.64	-	√	*
168	Phenylalanine	$C_9H_{11}NO_2$	171.1107	166.0863	166.0864	M+H	0.6	√	√	*
169	Methylxanthine	$C_6H_6N_4O_2$	166.0478	167.0554	167.0551	M+H	1.8	√	√	*
170	Methoxytyramine	$C_9H_{13}NO_2$	167.0946	168.1018	168.1019	M+H	0.77	-	√	
				185.1284	185.1284	M+NH4	0.15	-	√	
171	Dimethoxy-4-methylphenol	$C_9H_{12}O_3$	168.0786	169.0861	169.0859	M+H	0.92	√	√	
				186.1125	186.1124	M+NH4	0.3	√	√	
172	Oxodecanoic acid	$C_{10}H_{18}O_3$	186.1256	169.1222	169.1229	M+H-H2O	4.14	√	√	*
173	Dopamine	$C_8H_{11}NO_2$	153.079	171.1128	171.1128	M+NH4	0.13	-	√	
174	Dimethyl-2,5-heptadienoic acid	$C_9H_{14}O_2$	154.0994	172.1331	172.1332	M+NH4	0.46	√	√	*

No.	Metabolite name	Molecular formula	Exact mass	Obsmz	Theomz	Adduct	Error, ppm	nanoESI	nanoESI-APCI	LC-MS
				177.0883	177.0886	M+Na	1.58	√	√	
175	Glycerol 3-phosphate	$C_3H_9O_6P$	172.0137	173.0211	173.021	M+H	0.73	-	√	
176	Octenedioic acid	$C_8H_{12}O_4$	172.0736	173.081	173.0809	M+H	0.82	√	√	*
				190.1073	190.1074	$M+NH_4$	0.31	√	√	*
177	N-Acetyl-L-glutamate 5-semialdehyde	$C_7H_{11}NO_4$	173.0688	174.0762	174.0761	M+H	0.53	-	√	
178	cis-aconitate	$C_6H_6O_6$	174.0164	174.0841	174.084	M+·	0.57	-	√	
179	Diethylbenzeneacetamide	$C_{12}H_{17}NO$	191.131	174.1278	174.1283	$M+H-H_2O$	2.95	√	√	
				192.1382	192.1383	M+H	0.59	√	√	*
180	Dimethyl adipate	$C_8H_{14}O_4$	174.0892	175.0965	175.0965	M+H	0.05	√	√	
				192.1229	192.123	$M+NH_4$	0.57	√	√	
				197.0782	197.0784	M+Na	1.06	√	√	*
181	Hydroxynonanoic acid	$C_9H_{18}O_3$	174.1256	175.1327	175.1329	M+H	1.11	√	√	
				192.1594	192.1594	$M+NH_4$	0.03	-	√	
182	Glucosamine	$C_6H_{13}NO_5$	179.0794	180.0866	180.0867	M+H	0.4	√	√	*
183	Histidine trimethylbetaine	$C_9H_{15}N_3O_2$	197.1164	180.1145	180.1137	$M+H-H_2O$	4.29	-	√	*
184	Fucose	$C_6H_{12}O_5$	164.0685	182.1023	182.1023	$M+NH_4$	0.15	-	√	
185	Dodecenoic acid	$C_{12}H_{22}O_2$	198.162	181.1586	181.1593	$M+H-H_2O$	3.75	√	√	
				199.1691	199.1693	M+H	0.9	√	√	*
				216.1958	216.1958	$M+NH_4$	0.09	√	√	*
186	Methionine sulfoxide	$C_5H_{11}NO_3S$	165.046	183.0804	183.0798	$M+NH_4$	3.47	√	√	*

No.	Metabolite name	Molecular formula	Exact mass	Obsmz	Theomz	Adduct	Error, ppm	nanoESI	nanoESI-APCI	LC-MS
187	Decenedioic acid	$C_{10}H_{16}O_4$	200.1049	183.1019	183.1022	M+H-H_2O	1.41	√	√	*
188	9-Hydroxy-10-undecenoic acid	$C_{11}H_{20}O_3$	200.1412	183.1381	183.1385	M+H-H_2O	2.43	√	√	
				201.1484	201.1485	M+H	0.72	√	√	*
				218.1751	218.175	M+NH_4	0.25	√	√	
189	Dodecanoic acid	$C_{12}H_{24}O_2$	200.1776	183.1743	183.1749	M+H-H_2O	3.44	√	√	
				201.1848	201.1849	M+H	0.65	√	√	*
				218.2113	218.2114	M+NH_4	0.6	-	√	*
190	Phosphorylcholine	$C_5H_{15}NO_4P^+$	184.0733	184.0734	184.0733	M+	0.74	√	√	*
191	Methyprylon	$C_{10}H_{17}NO_2$	183.1259	184.1331	184.1332	M+H	0.43	√	√	*
192	Undecenoic Acid	$C_{11}H_{20}O_2$	184.1463	185.1536	185.1536	M+H	0.16	√	√	*
193	5-(2-Methylpropyl)tetrahydro-2-oxo-3-furancarboxylic acid	$C_9H_{14}O_4$	186.0892	187.0965	187.0965	M+H	0.05	√	√	
				209.0783	209.0784	M+Na	0.52	√	√	*
194	Dihydroxyphenylglycol	$C_8H_{10}O_4$	170.0579	188.0917	188.0917	M+NH_4	0.05	-	√	*
195	Arabinonic acid	$C_5H_{10}O_6$	166.0477	189.0372	189.0369	M+Na	1.39	√	√	
196	Ethyl 2,4-dimethyl-1,3-dioxolane-2-acetate	$C_9H_{16}O_4$	188.1049	189.112	189.1122	M+H	0.84	√	√	*
197	Acetyl-lysine	$C_8H_{16}N_2O_3$	188.1161	189.1232	189.1234	M+H	1.02	-	√	*
198	Trimethyl-L-lysine	$C_9H_{20}N_2O_2$	188.1525	189.1595	189.1598	M+H	1.47	-	√	*
199	Citric Acid	$C_6H_8O_7$	192.027	192.0838	192.0841	M+·	1.56	√	√	
200	3-Hydroxy-5-phenylpentanoic acid	$C_{11}H_{14}O_3$	194.0943	195.1015	195.1016	M+H	0.48	√	√	*
				212.1279	212.1281	M+NH_4	0.92	-	√	*

No.	Metabolite name	Molecular formula	Exact mass	Obsmz	Theomz	Adduct	Error, ppm	nanoESI	nanoESI-APCI	LC-MS
201	2-Carboxy-5,7-dimethyl-4-octanolide	$C_{11}H_{18}O_4$	214.1205	197.1171	197.1178	M+H-H_2O	3.6	√	√	
				215.1273	215.1278	M+H	2.37	√	√	
202	6-(3,4-Methylenedioxyphenyl)-3,5-hexadien-2-one	$C_{13}H_{12}O_3$	216.0786	199.0752	199.0759	M+H-H_2O	3.74	√	√	*
203	Acetylcarnitine	$C_9H_{17}NO_4$	203.1158	204.123	204.1231	M+H	0.28	√	√	*
204	5-(3E-Pentenyl)tetrahydro-2-oxo-3-furancarboxylic acid	$C_{10}H_{14}O_4$	198.0892	199.0963	199.0965	M+H	1.05	√	√	
				216.1229	216.123	M+NH_4	0.5	√	√	
205	5-Pentyltetrahydro-2-oxo-3-furancarboxylic acid	$C_{10}H_{16}O_4$	200.1049	201.112	201.1122	M+H	0.79	√	√	*
206	7-Methyl-3-methylene-1,2,6,7-octanetetrol	$C_{10}H_{20}O_4$	204.1362	205.1433	205.1435	M+H	0.78	√	√	
				222.1699	222.17	M+NH_4	0.27	√	√	
207	Thymidine	$C_{10}H_{14}N_2O_5$	242.0903	207.078	207.0776	M+H-2H_2O	2.07	√	√	
208	Phenylalanine betaine	$C_{12}H_{17}NO_2$	207.1259	208.1331	208.1332	M+H	0.62	√	√	*
209	Myristic acid	$C_{14}H_{28}O_2$	228.2089	211.2054	211.2062	M+H-H_2O	3.93	√	√	*
				229.2161	229.2162	M+H	0.57	√	√	
210	Myristoleic acid	$C_{14}H_{26}O_2$	226.1933	209.1898	209.1906	M+H-H_2O	3.73	√	√	*
				227.2005	227.2006	M+H	0.35	√	√	*
				244.227	244.2271	M+NH_4	0.33	√	√	
211	Heptanoyl-homoserine lactone	$C_{11}H_{19}NO_3$	213.1364	214.1436	214.1437	M+H	0.47	-	√	
212	Propionylcarnitine	$C_{10}H_{19}NO_4$	217.1314	218.1385	218.1387	M+H	0.95	√	√	*
213	Pantothenic acid	$C_9H_{17}NO_5$	219.111	220.1179	220.1183	M+H	1.82	√	√	*
214	Asymmetric dimethylarginine	$C_8H_{18}N_4O_2$	202.143	220.177	220.1768	M+NH_4	1.02	√	√	*

No.	Metabolite name	Molecular formula	Exact mass	Obsmz	Theomz	Adduct	Error, ppm	nanoESI	nanoESI-APCI	LC-MS
215	Vanillylmandelic acid	$C_9H_{10}O_5$	198.0528	221.0421	221.042	M+Na	0.35	-	√	
216	Pentadecanoic acid	$C_{15}H_{30}O_2$	242.2246	225.2212	225.2219	M+H-H_2O	3.02	√	√	
				243.2318	243.2319	M+H	0.33	√	√	*
				260.2582	260.2584	M+NH_4	0.69	-	√	
217	Hydroxydodecanedioic acid	$C_{12}H_{22}O_5$	246.1467	229.1433	229.144	M+H-H_2O	3.16	√	√	*
218	Acylcarnitine C4:1	$C_{11}H_{19}NO_4$	229.1325	230.1386	230.1397	M+H	4.78	-	√	*
219	Butyrylcarnitine	$C_{11}H_{21}NO_4$	231.1471	232.1543	232.1543	M+H	0	√	√	*
220	Hexadecadienoic acid	$C_{16}H_{28}O_2$	252.2089	235.2056	235.2062	M+H-H_2O	2.68	√	√	
				270.2426	270.2427	M+NH_4	0.48	-	√	*
221	Palmitic acid	$C_{16}H_{32}O_2$	256.2402	239.2367	239.2375	M+H-H_2O	3.47	√	√	*
				257.2471	257.2475	M+H	1.67	√	√	*
				274.2738	274.274	M+NH_4	0.84	√	√	*
222	Acylcarnitine C5:1	$C_{12}H_{21}NO_4$	243.1482	244.1542	244.1554	M+H	4.91	-	√	*
223	Acylcarnitine C5:0	$C_{12}H_{23}NO_4$	245.1628	246.1699	246.17	M+H	0.41	√	√	*
224	Acylcarnitine C4-OH	$C_{11}H_{21}NO_5$	247.1421	248.1491	248.1493	M+H	0.81	-	√	*
225	Didesmethylvenlafaxine	$C_{15}H_{23}NO_2$	249.1729	250.18	250.1802	M+H	0.72	-	√	*
226	Dodecanedioic aicd	$C_{12}H_{22}O_4$	230.1518	253.1408	253.141	M+Na	0.83	√	√	*
227	Palmitoleic acid	$C_{16}H_{30}O_2$	254.2237	255.2317	255.231	M+H	2.74	√	√	*
228	Decanoylhomoserine	$C_{14}H_{25}NO_3$	255.1839	256.1905	256.1912	M+H	2.73	-	√	*
229	Acylcarnitine C6:1	$C_{13}H_{23}NO_4$	257.1639	258.1699	258.1711	M+H	4.65	-	√	*
230	Methyluridine	$C_{10}H_{14}N_2O_6$	258.0852	259.094	259.0925	M+H	5.84	-	√	*

No.	Metabolite name	Molecular formula	Exact mass	Obsmz	Theomz	Adduct	Error, ppm	nanoESI	nanoESI-APCI	LC-MS
231	Glu-Leu	$C_{11}H_{20}N_2O_5$	260.1384	261.1459	261.1457	M+H	0.77	√	√	*
232	Acylcarnitine C5-OH	$C_{12}H_{23}NO_5$	261.1577	262.1648	262.1645	M+H	1.14	√	√	*
233	Stearic acid	$C_{18}H_{36}O_2$	284.2715	267.2678	267.2688	M+H-H_2O	3.86	-	√	
				285.2785	285.2788	M+H	1.16	√	√	*
234	Acylcarnitine C7:0	$C_{14}H_{27}NO_4$	273.1942	274.2012	274.2014	M+H	0.73	-	√	*
235	Acylcarnitine C6-OH	$C_{13}H_{25}NO_5$	275.1735	276.1805	276.1807	M+H	0.72	-	√	*
236	Ethyl-3-hydroxybutyrate glucoside	$C_{12}H_{22}O_8$	294.1315	277.1279	277.1288	M+H-H_2O	3.13	√	√	
237	Stearidonic acid	$C_{18}H_{28}O_2$	276.2089	277.2158	277.2162	M+H	1.55	-	√	*
238	Vaccenic acid	$C_{18}H_{34}O_2$	282.2559	283.2631	283.2632	M+H	0.28	-	√	*
239	Acylcarnitine C8:0	$C_{15}H_{29}NO_4$	287.2099	288.2167	288.2171	M+H	1.39	-	√	*
240	Acylcarnitine C7-OH	$C_{14}H_{27}NO_5$	289.1892	290.196	290.1964	M+H	1.38	-	√	*
241	Sphingosine	$C_{18}H_{37}NO_2$	299.2824	300.2895	300.2897	M+H	0.76	-	√	*
242	Dimethylheptanoyl carnitine	$C_{16}H_{31}NO_4$	301.2253	302.2323	302.2326	M+H	1.02	-	√	*
243	Dihydrosphingosine	$C_{18}H_{39}NO_2$	301.2981	302.3052	302.3054	M+H	0.59	√	√	*
244	Acylcarnitine C8-OH	$C_{15}H_{29}NO_5$	303.2049	304.2116	304.2121	M+H	1.64	-	√	*
245	4,8-Dimethylnonanoyl carnitine	$C_{18}H_{35}NO_4$	329.2566	312.2531	312.2539	M+H-H_2O	2.59	-	√	
				330.2634	330.2639	M+H	1.54	-	√	*
246	Acylcarnitine C10:0	$C_{17}H_{33}NO_4$	315.2413	316.248	316.2485	M+H	1.58	-	√	*
247	α-CEHC	$C_{16}H_{22}O_4$	278.1518	317.1148	317.115	M+K	0.63	√	√	*
248	Icosatetraenoic acid	$C_{20}H_{32}O_2$	304.2402	322.2739	322.274	M+NH_4	0.4	-	√	*

No.	Metabolite name	Molecular formula	Exact mass	Obsmz	Theomz	Adduct	Error, ppm	nanoESI	nanoESI-APCI	LC-MS
249	γ-linolenyl carnitine	$C_{25}H_{43}NO_4$	421.3192	422.3259	422.3265	M+H	1.44	-	√	
250	Palmitoylcarnitine	$C_{23}H_{46}NO_4$	400.3421	423.3293	423.3313	M+Na	4.81	-	√	*
251	Glycochenodeoxycholic acid	$C_{26}H_{43}NO_5$	449.3141	450.321	450.3214	M+H	0.94	-	√	*
252	Deoxycytidine	$C_9H_{13}N_3O_4$	227.0906	455.1881	455.1885	2M+H	0.9	-	√	*
253	Glucose	$C_6H_{12}O_6$	180.0634	181.071	181.0707	M+H	1.72	√	-	*
				198.0973	198.0972	$M+NH_4$	0.56	−	√	
				203.0527	203.0526	M+Na	0.55	−	√	
254	2-Piperidinone	C_5H_6O	99.0684	100.0755	100.0757	M+H	1.65	√	√	*
255	Hydroxyphenylpyruvic acid	$C_9H_8O_4$	180.0423	181.0495	181.0496	M+H	0.32	√	-	
256	Tyrosine	$C_9H_{11}NO_3$	181.0739	182.0812	182.0812	M+H	0.04	√	-	*
257	Acetylspermidine	$C_9H_{21}N_3O$	187.1685	188.1757	188.1758	M+H	0.33	√	-	*
258	Spermine	$C_{10}H_{26}N_4$	202.2157	203.223	203.223	M+H	0.23	√	-	*
259	Glutathione	$C_{10}H_{17}N_3O_6S$	307.0838	308.0907	308.0911	M+H	1.32	√	-	*
				330.0728	330.073	M+Na	0.62	√	-	*

Table S4. Performance comparison between related studies and ours.

Study	Technology	LOD	Sample type	Metabolites number
Bao et al.[6]	PENG-ESI	100 pg/g	Pesticide	N/A
Cheng et al.[7]	Dual ESI+APCI source	N/A	Chemical and biological compounds, diesel, rosemary oils	N/A
Kulyk et al.[4]	Dual noncontact nESI/nAPCI source	12 pg/mL	Biofluids	N/A
Liu et al.[5]	Hybrid nanoESI-DBDI source	N/A	Single cells	111
Ours	Concentric hybrid nanoESI-APCI source	10 pg/mL	Single cells	258

References

[1] a) J. F. Cahill, V. Kertesz, G. J. Van Berkel, *Anal. Chem.* **2015**, *87* (21), 11113; b) M. R. EmmertBuck, R. F. Bonner, P. D. Smith, R. F. Chuaqui, Z. P. Zhuang, S. R. Goldstein, R. A. Weiss, L. A. Liotta, *Science* **1996**, *274* (5289), 998; c) R. F. Bonner, M. EmmertBuck, K. Cole, T. Pohida, R. Chuaqui, S. Goldstein, L. A. Liotta, *Science* **1997**, *278* (5342), 1481

[2] a) S. F. Macha, T. D. McCarley, P. A. Limbach, *Anal. Chim. Acta* **1999**, *397* (1-3), 235; b) J. Fang, H. Zhao, Y. Zhang, M. Lu, Z. Cai, *Trends in Environmental Analytical Chemistry* **2020**, *25*, ; c) J.-C. Wolf, L. Gyr, M. F. Mirabelli, M. Schaer, P. Siegenthaler, R. Zenobi, *J. Am. Soc. Mass. Spectrom.* **2016**, *27* (9), 1468; d) A. Good, D. A. Durden, P. Kebarle, *J. Chem. Phys.* **1970**, *52* (1), 212

[3] a) R. Gonzalez-Mendez, P. Watts, D. Olivenza-Leon, D. F. Reich, S. J. Mullock, C. A. Corlett, S. Cairns, P. Hickey, M. Brookes, C. A. Mayhew, *Anal. Chem.* **2016**, *88* (21), 10624; b) B. Yuan, A. R. Koss, C. Warneke, M. Coggon, K. Sekimoto, J. A. de Gouw, *Chem. Rev.* **2017**, *117* (21), 13187

[4] D. S. Kulyk, D. J. Swiner, T. Sahraeian, A. K. Badu-Tawiah, *Anal. Chem.* **2019**, *91* (18), 11562

[5] Q. Liu, J. Lan, R. Wu, A. Begley, W. Ge, R. Zenobi, *Anal. Chem.* **2022**, *94* (6), 2873

[6] M. Bao, J. Bai, Y. Wang, S. Zhu, Y. Liu, T. Wen, J. Zhang, S.-c. Ma, Y. Guo, *Anal. Chem.* **2023**, *95* (40), 14842

[7] S.-C. Cheng, S.-S. Jhang, M.-Z. Huang, J. Shiea, *Anal. Chem.* **2015**, *87* (3), 1743
